# Topical Application of Temperature-Sensitive Gel Containing Caerin 1.1 and 1.9 Peptides on TC-1 Tumour-Bearing Mice Induced High-Level Immune Response in the Tumour Microenvironment

**DOI:** 10.3389/fonc.2021.754770

**Published:** 2021-11-11

**Authors:** Guoying Ni, Xiaosong Liu, Hejie Li, Conor E. Fogarty, Shu Chen, Pingping Zhang, Ying Liu, Xiaolian Wu, Ming Q. Wei, Guoqiang Chen, Ping Zhang, Tianfang Wang

**Affiliations:** ^1^ Cancer Research Institute, First People’s Hospital of Foshan, Foshan, China; ^2^ Genecology Research Centre, University of the Sunshine Coast, Maroochydore DC, QLD, Australia; ^3^ Menzies Health Institute Queensland, Griffith University, Gold Coast, QLD, Australia; ^4^ The First Affiliated Hospital/School of Clinical Medicine of Guangdong Pharmaceutical University , Guangzhou, China; ^5^ School of Science, Technology and Engineering, University of the Sunshine Coast, Maroochydore DC, QLD, Australia

**Keywords:** TC-1 tumour, single cell RNA sequencing, caerin peptide, quantitative proteomics, tumour microenvironment

## Abstract

The development of topical cream drugs that increase the immune activation of tumour-infiltrating lymphocytes against tumour and chronic viral infection-associated lesions is of great immunotherapeutic significance. This study demonstrates that the topical application of a temperature-sensitive gel containing caerin 1.1 and 1.9 peptides reduces nearly 50% of the tumour weight of HPV16 E6/E7-transformed TC-1 tumour-bearing mice *via* improving the tumour microenvironment. Confocal microscopy confirms the time-dependent penetration of caerin 1.9 through the epidermal layer of the ear skin structure of mice. Single-cell transcriptomic analysis shows that the caerin 1.1/1.9 gel expands the populations with high immune activation level and largely stimulates the pro-inflammatory activity of NK and dendritic cells. Closely associated with INFα response, *Cebpb* seems to play a key role in altering the function of all *Arg1^hi^* macrophages in the caerin group. In addition, the caerin gel treatment recruits almost two-fold more activated CD8^+^ T cells to the TME, relative to the untreated tumour, which shows a synergistic effect derived from the regulation of S1pr1, *Ccr7*, *Ms4a4b* and *Gimap* family expression. The TMT10plex-labelling proteomic quantification further demonstrates the activation of interferon-alpha/beta secretion and response to cytokine stimulus by the caerin gel, while the protein contents of several key regulators were elevated by more than 30%, such as *Cd5l*, *Gzma*, *Ifit1*, *Irf9* and *Stat1*. Computational integration of the proteome with the single-cell transcriptome consistently suggested greater activation of NK and T cells with the topical application of caerin peptide gel.

## Introduction

Among 14 million new cancer cases reported worldwide in 2012, human papillomavirus (HPV) infection-associated cancers accounted for 4.6% of total cancers and nearly 30% of infection-related cancers (1). High-risk HPV infection is related to a fraction of head-and-neck epithelial carcinoma in both developed and undeveloped countries (2), whereas the linking between HPV with cancers of the anus, vulva, vagina and penis is evident. Genital warts (condyloma acuminate, venereal warts and anogenital warts) are one of the most common sexually transmitted diseases (STDs) resulting from infection with low-risk HPV, especially HPV6 and 11, which lead to approximately 90% of the cases (3–5).

The introduction of a prophylactic vaccine against HPV infection has greatly reduced the incidence of genital warts (6–8). However, the prevalence of anogenital warts in the USA has risen for the past 35 years (9). Topical application of 5% imiquimod cream (Aldara, Loughborough, UK), podophyllotoxin or sinecatechin/polyphenon E is recommended as a first-line treatment for genital warts, with subsequent physical or chemical ablation recommended for larger warts (5, 10). Imiquimod is known to induce the secretion of proinflammatory cytokines (11), including interferon-alpha (12). However, there are significant side effects associated with the topical application of imiquimod, such as erythema, scabbing, itching and burning (13). Additionally, imiquimod-associated adverse effects at non-application sites were reported, such as fever, vertigo or myalgia, as well as distant inflammatory mucosal reactions (14). Thus, alternative treatments with minimal side effects have been under investigation.

Many host-defence peptides discovered from skin secretion of different Anura species show broad-spectrum antibacterial and antifungal activities, and the ability to permeabilise mammalian cells (15). In addition, immunomodulatory, chemoattractant and insulinotropic properties have been characterised from a number of host-defence peptides, making them potent anticancer agents (16–18). It was postulated that certain cationic α-helical peptides executed their anticancer activity by disruption of the plasma membrane, while some others induced apoptosis *via* the modulation of the key mitochondrial pathway and the binding of the peptides to specific cell surface receptors to subsequently facilitate entry into the cytoplasm was implicated (19). Isolated from the epidermal secretion of Australian amphibians, *Litoria genus*, caerin 1.1 and 1.9 peptides were found to significantly inhibit the proliferation of TC-1 (20, 21) and HeLa cells (22) at the concentrations non-toxic to typical cells. They appeared to stimulate the signalling of TNFα-mediated apoptosis and activate the TCR pathway in HeLa cells (22, 23). Caerin 1.1 and 1.9 largely inhibited the growth of TC-1 tumour in mice, and the inhibition required an intact adaptive immune system; in addition, the treatment prolonged the survival time of vaccinated and PD-1 blocked TC-1 tumour-bearing mice significantly (23). However, the detailed molecular mechanism underpinning the tumour suppressive effect induced by these caerin peptides remains elusive.

Previously, we have developed a temperature-sensitive caerin 1.1 and 1.9 gel (liquid at 4°C–35°C, solid at 37°C). The caerin 1.1 and 1.9 gel, but not the control gel, was able to inhibit TC-1 and HeLa cell proliferation *in vitro*. Moreover, the caerin 1.1 and 1.9 gel inhibited TC-1 tumour growth *in vivo*, either through direct injection or, more interestingly, through topical application to subcutaneously transplanted TC-1 tumour (21). In this study, tumour-infiltrating hematopoietic cells isolated from TC-1 tumour-bearing mice treated with the caerin peptide gel were subjected to scRNA-seq analysis, to reveal the modulation of the tumour-infiltrating cell landscape in the tumour microenvironment (TME) post the treatment. A mass spectrometry-guided quantitative proteomic analysis was performed to investigate the effect of the treatment on the TME at the protein level. Our study provides new insights into the heterogeneity of tumour-infiltrating cells and identifies novel markers to define immune-activating macrophages and dendritic cells. Moreover, the alteration of the developmental process of NK cells and the recruitment of more activated CD8^+^ T cells due to the caerin gel treatment are revealed.

## Results

### Topical Application of the Caerin Gel Inhibited the Growth of TC-1 Tumour

**Figure 1:**
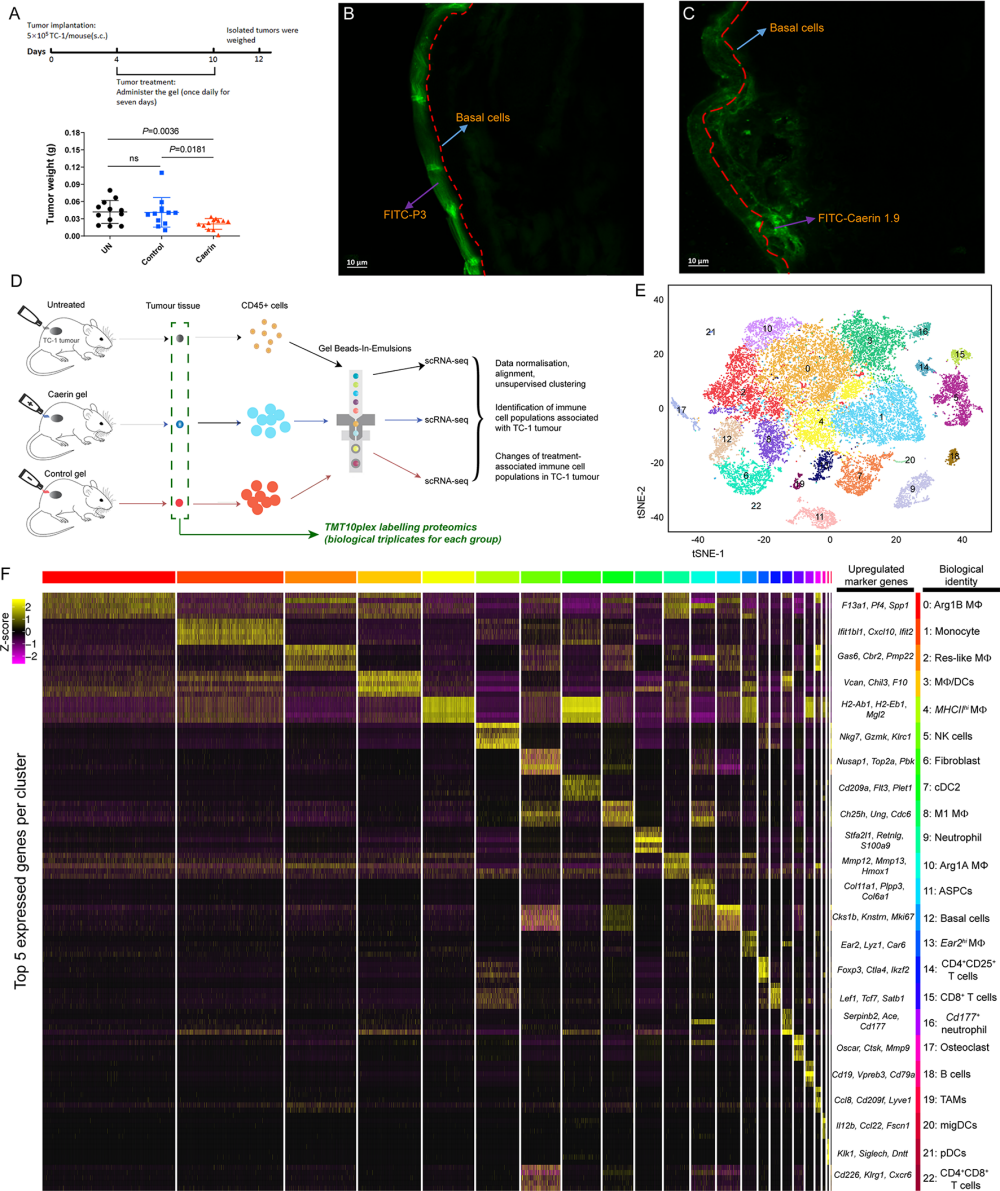
Topical application of caerin 1.1/1.9 gel on TC-1 tumour-bearing mice and the identification of immune cell populations by single-cell RNA sequencing. **(A)** Timeline of topical application of the gels on the tumours of TC-1-bearing mice (top). Topical application of caerin 1.1/1.9 gel which inhibited TC-1 tumour growth (bottom). C57/BL6 mice were subcutaneously transplanted with 5 × 10^5^ TC-1 tumour cells. Three days post transplantation, the tumour areas of five mice per group were treated topically with either control peptide (P3) gel or caerin 1.1/1.9 gel daily for 7 days. Mice were sacrificed 2 days after the final treatment for analysis of tumour weights. Data represent the tumour weights of individual mice, and the mean is shown. ns, not significant. Fluorescence microscopy of the penetrations of caerin 1.9 **(B)** or control peptide **(C)** containing gel at 5 min post the topical application on TC-1 tumour. Five microliters of 1 µg/µl FITC-labelled caerin 1.9 or control peptide was used. **(D)** Schematic diagram of the experimental design (single-cell RNA seq and quantitative proteomics) and data processing. **(E)** t-Stochastic neighbour embedding (t-SNE) representation of aligned gene expression data in single cells extracted from the untreated, the topical application of the gel containing caerin 1.1/1.9 or control of TC-1 bearing mice showing partition into 23 distinct clusters. **(F)** Selected enriched genes used for biological identification of each cluster (scale: log2 fold change). MΦ represents macrophage; ASPC, adipogenic stem and precursor cell; NK, natural killer cells; cDC, conventional dendritic cell; migDC, migratory dendritic cell; pDC, plasmacytoid dendritic cell; and TAM, tumour-associated macrophage (see **Supplementary Data 2** for the full list of all marker genes detected).

Tumour-bearing mice were topically treated with the gel containing either caerin 1.1 and 1.9 peptides (molar ratio 1:1; “caerin”) or a control peptide P3 (“control”) (**Figure 1A**, top). The tumour weights were significantly reduced in the caerin group (**Figure 1A**, bottom). To investigate the mechanism underlying this antitumor activity, the penetration of caerin 1.9 through the skin was assessed using confocal microscopy. At 5 min post the topical application, FITC-labelled P3 was mostly distributed on the epidermal layer (**Figure 1B**), while FITC-labelled caerin 1.9 was largely present beneath the basal cell layer with a significantly in-depth distribution (**Figure 1C** and **Figure S1**). Additionally, the dynamic trace of green fluorescence in the cross section of the tissues showed that FITC-labelled caerin 1.9 penetrated to more areas under the basal cell layer (**Supplementary V1, V2**). These results confirm our previous observation that caerin 1.1 and 1.9 are able to penetrate intact skin and lead to the growth inhibition of subcutaneously transplanted TC-1 cells (21).

### Single-Cell RNA-Seq Revealed Complex Heterogeneity of Non-Macrophage Cells in the TME

Total viable CD45^+^ leukocytes were isolated from the untreated, caerin and control groups (**Figure 1D** and **Supplementary Data 1**). The unsupervised graph-based clustering method detected a total of 23 distinct cell clusters (cluster “0” to “22”) (**Figures 1E, F** and **Figures S3, S4**), and their identities were annotated based on the marker genes (**Supplementary File 1**). The gene expression data from extracted CD45^+^ cells were aligned and projected in a two-dimensional space through t-stochastic neighbour embedding (t-SNE) for the identification of tumour-associated immune cell populations and the differentially expressed genes associated with different groups (**Figure S2**). The presence of established canonical marker genes, such as *Nkg7*, *Cd19*, *Fcmr*, *Cd8b1* and *Cd79a*, indicated the identities of lymphocyte lineages. Myeloid cells were supported by the identification of *Cd209a*, *Adgre1*, *Itgax*, *Csf1r*, *Lgals3*, *Cd11c*, *Cd14*, *Cd68*, *Ccr2* and *Ly6c2* (24, 25) (**Supplementary Data 2**). The expressions of the top 5 marker genes of each cluster were compared, showing a relatively high overlap between clusters 0 and 5 with other clusters, respectively (**Figure S4**).

Non-macrophage cells included high populations of monocytes (cluster 1; marker genes: *Ly6a*, *Ly6c2*, *Fcgr1* and *Dpep2*) and natural killer (NK) cells (cluster 5; *Nkg7*, *Gzmk*, *Klrc1* and *Cxcr6*) (**Figure 1F**). Two clusters were detected as neutrophils, i.e., cluster 9 (*Stfa2l1*, *Retnlg*, *S100a9* and *Asprv1*) and cluster 16 (*Serpinb2*, *Ace*, *Cd177* and Ifitm6). B cells (cluster 18; *Cd19*, *Cd79a*, *Fcmr* and *Vperb3*) had a small population. Three populations showed the signature of dendritic cells, including conventional DC type 2 (cDC2) (cluster 7; *Plet1*, *Cd209a*, *Ctnnd2* and *Epcam*) (26), migratory DCs (migDC) (cluster 20; *Ccl22*, *Bcl2l14*, *Fscn1* and *Cacnb3*) (27) and plasmacytoid dendritic cells (pDCs) (cluster 21; *Siglech*, *Ccr9*, *Ly6d* and *Pacsin1*) (26). Moreover, there were three clusters with gene signatures characterising the phenotypes of T cells (clusters 14, 15 and 22). Cluster 14 was assigned to CD4^+^CD25^+^ T cells, represented by *Foxp3*, *Ctla4*, *Ikzf2* and *Tnfrsf4*, while cluster 15 corresponded to CD8^+^ T cells with the signatures of *Lef1*, *Tcf7*, *Satb1* and *Cd8b1*. CD4^+^CD8^+^ T cells had the lowest number of cells, with the marker genes such as *Cd226*, *Klrg1* and *Cxcr6*. Fibroblast (cluster 6; *Nusap1*, *Top2a*, *Pclaf* and *Mki67*) (28), adipogenic stem and precursor cells (ASPCs) (cluster 11; *Col11a1*, *Plpp3*, *Col6a1* and *Gas1*) (23, 29), basal cells (cluster 12; *Ccnb2*, *Cdkn3*, *Hmmr* and *Birc5*) (30) and osteoclast (Cluster 17; *Oscar*, *Ctsk* and *Mmp9*) (31) were detected as possible contaminants (also see **Supplementary Data 2**).

### Caerin Gel Modulated the Functions of *Arg1^hi^* Tumour-Infiltrating Macrophages to Be More Immune Active

A total of eight MΦ populations were present. The marker genes of cluster 0, including *Pf4*, *Arg1*, *Pdpn* and *F13a1*, were used to characterise the Arg1B MΦ in a previous study (32) (**Supplementary Data 2**). Cluster 2 corresponded to a resident-like MΦ due to the high expression of *Gas6*, *Stab1*, *Mrc1* and *Folr2* (33). Cluster 3 exhibited mixed cell phenotypes, including proinflammatory MΦ (*Cxcl10*, *Gbp2* and *Thbs1*), *Ly6c^hi^* infiltrating MΦ (*Chil3* and *Plac8*) and dendritic cells (*Rsad2*, *Ifit1*, *Ifit2* and *Ifi205*). Thus, this cluster was labelled as MΦ/DCs. The significantly high expressions of *MHCII* members, such as *H2-Ab1*, *H2-Aa*, *H2-Eb1* and *H2-DMb1I*, together with *Cd74*, suggested cluster 4 with an *MHCII^hi^* MΦ phenotype (27). Cluster 8 showed marker genes depicting M1 MΦ-like phenotypes, including *Ccl12* (34) and *Cx3cr1* (35), as well as many genes playing roles in cell growth. Arg1A MΦ was assigned to cluster 10, with the signatures *Arg1*, *Mmp12*, *Mmp13* and *Lgals3* (32). The marker gene *Ear2* was exclusive to cluster 13; it was thus assigned as *Ear2^hi^* MΦ (36, 37). Cluster 19 was characterised by MΦ markers such as *Lyve1*, *Cd209f* and *Cd163*, while the marker gene *Ccl8* was previously identified as the signature for TAM (38).

The sum of these MΦs represented the largest cell population, constituting 55.14% of the total cells in the untreated TC-1 tumour, and a similar fraction in mice treated with caerin (56.39%) or control gel (55.86%) (**Supplementary Data 2**). It appeared that, in comparison to the control group, the caerin gel largely increased the proportions of Res-like MΦ (by 12.1%) and Arg1A MΦ (25.1%) (**Supplementary Data 1** and **Figure S3C**). Notably, the population of Arg1A MΦ increased by 23.1% in the caerin group compared to the untreated group. The enrichment of TNFα signalling *via* NF-κB and IL-6/JAK/STAT3 signalling in the *Arg1^hi^* MΦs of the control or untreated group, relative to the caerin group, was identified. Both pathways have been found to suppress the anti-tumour immune response in the TME, *via* enhancing proliferation, survival, invasiveness and metastasis of the tumour cells (39, 40). This suggested that the caerin gel reduced the immunosuppression of *Arg1^hi^* MΦs in the TME of TC-1 tumour.

**Figure 2:**
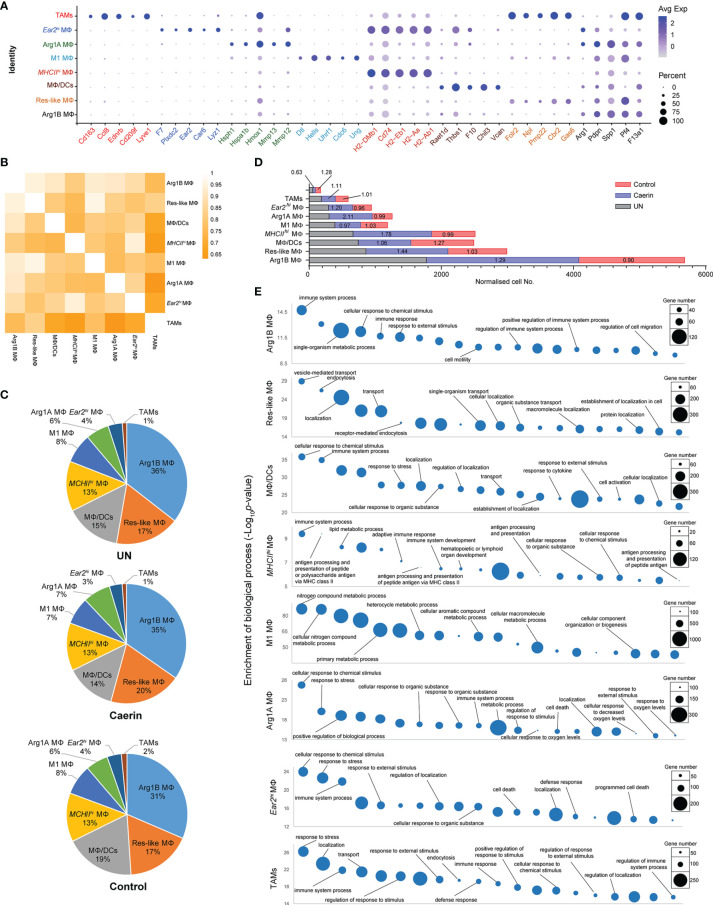
The modulation of the heterogeneity of macrophages in TC-1 tumour with the topical application of gels. **(A)** Bubble map of top 5 marker gene expression in different macrophage populations, including Arg1B MΦ, Res-like MΦ, MΦ/DCs, *MHCII^hi^* MΦ, M1 MΦ, Arg1A MΦ, *Ear2^hi^* MΦ and TAM. The bubble size represents the ratio of the sum of the expression levels of the marker genes in a certain population to the sum of their expression levels in all cells, while the bubble colour represents the average expression of the marker genes in the cell population. **(B)** Correlation analysis among nine MΦ populations based on the expressions of marker genes. **(C)** The proportions of different macrophages in untreated, caerin 1.1/1.9 gel and control. **(D)** The comparison of the normalised expression of selected marker genes across nine macrophage populations. **(E)** Gene ontology enrichment analysis of biological processes in eight MΦ populations in the TC-1 tumour. The top 20 enriched biological processes were compared in terms of *p*-value and gene numbers, respectively.

The average expression of the top five marker genes of each MΦ population was compared with respect to other genes across all MΦ cell populations (**Figure 2A**). The signature of *Ear2^hi^* MΦ and TAMs appeared more exclusive, while the marker genes of Arg1B and *MHCII^hi^* MΦ showed certain expressions in other MΦs. The correlation among these MΦs based on the expression of significantly upregulated genes demonstrated that TAMs correlated least with Arg1B, *Ear2^hi^* and *MHCII^hi^* MΦs (**Figure 2B**). Res-like MΦ was highly correlated with M1 MΦ, as were *Ear2^hi^* with *MHCII^hi^* MΦs. The proportions of the eight MΦs appeared similar in different groups (**Figure 2C**). However, the comparison of normalised cell numbers found significant elevations in Arg1B (fold change, FC = 1.29), Res-like (FC = 1.44), *MHCII^hi^* (FC = 1.78) and Arg1A MΦs (FC = 2.11) in the caerin group, while TAMs were largely reduced to 0.63 and 0.47 with reference to the untreated or control group, respectively (**Figure 2D**). The top 20 enriched biological processes of these MΦ populations were compared (**Figure 2E** and **Supplementary Data 3**). Immune response relevant processes, such as immune system process, cellular response to chemical stimulus and response to external stimulus, were highly detected in all MΦs, except Res-like and M1 MΦs. *MHCII^hi^* MΦ played a considerable role in professional antigen processing and presenting.

**Figure 3:**
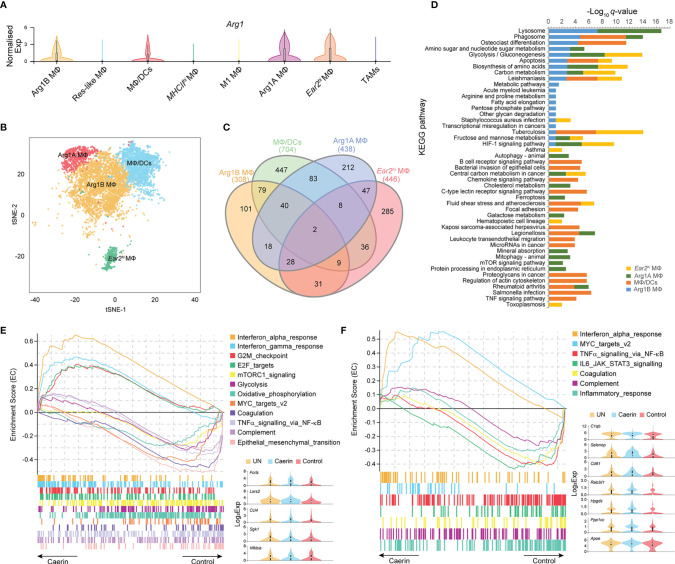
Caerin 1.1/1.9 containing gel modulated the function of *Arg1^hi^* macrophages. **(A)** Comparison of the normalised expression of *Arg1* in eight MΦ populations. **(B)** 2D t-SNE distributions of *Arg1^hi^* MΦs, including Arg1B, Arg1A, MΦ/DCs and *Ear2^hi^* MΦs. **(C)** Venn diagram compares all the marker genes identified in *Arg1^hi^* MΦs. **(D)** Comparison of KEGG pathways enriched (*q*-value < 0.05) in *Arg1^hi^* MΦs. **(E)** GSEA reveals that enrichment of interferon *α* and *γ* responses, G2M checkpoint and E2F targets gene sets in Arg1B MΦs of the caerin group compared to the control group. **(F)** GSEA reveals that enrichment of MYC targets v2 and interferon *α* response gene sets in Arg1A MΦs of the caerin group compared to the control group. The expressions (Log_2_ value) of the genes upregulated significantly in Arg1B or Arg1A MΦs of the caerin group in comparison to both the untreated and control groups were displayed, respectively (see **Figure S5** for the GSEA analysis of MΦ/DCs and *Ear2^hi^* MΦs and **Supplementary Data 4** for detailed results).

*Arg1^hi^* MΦs are known to deplete L-arginine locally, to assist wound healing and tissue fibrosis, and have been considered immunosuppressive and tumorigenic in certain tumours (41, 42). *Arg1* was highly expressed in four MΦ clusters, including MΦ/DCs, Arg1A, Arg1B and *Ear2^hi^* MΦs (**Figure 3A**). Projected to a two-dimensional tSNE space, *Ear2^hi^* MΦ was distributed more separately from the other three MΦs, indicating possible functional specificity (**Figure 3B**). MΦ/DCs had the highest number of total (704) and unique (447) genes significantly upregulated (**Figure 3C**). The KEGG pathway analysis revealed that apoptosis was highly enriched in MΦ/DCs, Arg1B and *Ear2^hi^* MΦs (*q*-value < 0.0017) (**Figure 3D** and **Supplementary Data 3**). MΦ/DCs were more enriched with immune response relevant pathways than other MΦs, such as chemokine signalling, B cell receptor and TNF signalling. Ferroptosis, autophagy and mitophagy pathways were detected with significance only in Arg1A MΦ, while *Ear2^hi^* MΦ was comparatively more enriched with HIF-1 signalling and glycolysis/gluconeogenesis.

The GSEA analysis found that interferon *α* and *γ* responses, G2M checkpoint and E2F targets were most enriched in Arg1B MΦs of the caerin group, whereas glycolysis, oxidative phosphorylation, TNFα signalling *via* NF-κB and epithelial–mesenchymal transition were most enriched in that of the control group (**Figure 3E** and **Supplementary Data 4**). Five macrophage-associated genes, *Fcrls*, *Lars2*, *Ccl4*, *Sgk1* and *Nfkbia*, were upregulated (adj. *p* < 0.05) in the caerin group. MYC targets v2 and INF*α* response became more pronounced in Arg1A MΦs (**Figure 3F**), while the latter was also enriched in MΦ/DCs (**Figure S5A**). The EC value of inflammatory response was similar for the caerin and control groups. INF*α* response was the only pathway enriched in *Ear2^hi^* MΦ by the caerin gel (**Figure S5B**). Notably, the expression of *Cebpb* was remarkably upregulated in MΦ/DCs and *Ear2^hi^* MΦ of the caerin group.

### Caerin Gel Altered the Heterogeneity and Function of Dendritic Cells

**Figure 4:**
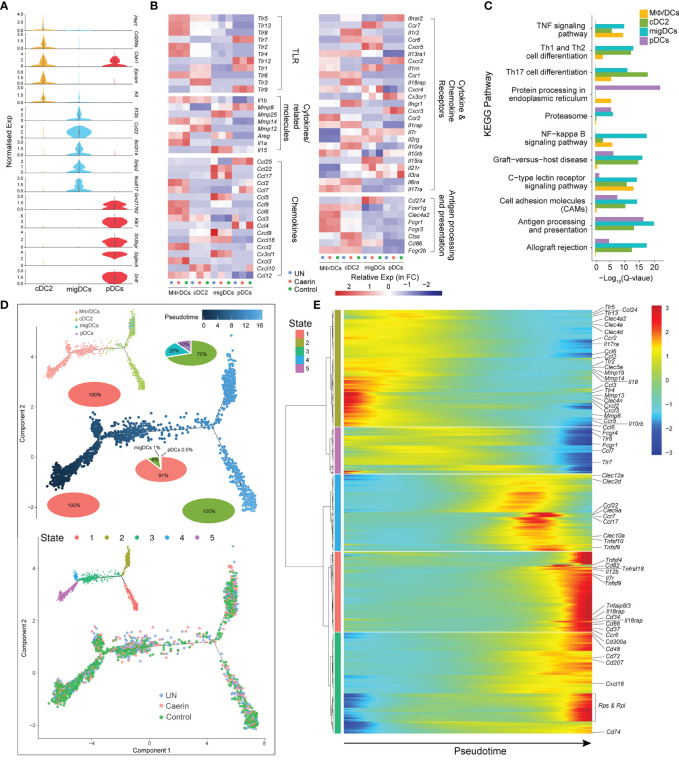
Caerin 1.1/1.9 gel induced the function variations in DCs. **(A)** Comparison of the normalised expression of the marker genes in cDC2, migDCs and pDCs, including *Dntt*, *Siglech*, *Sh3bgr*, *Klk1*, *Gm21762*, *Nudt17*, *Strip2*, *Bcl2l14*, *Ccl22*, *Il12b*, *Kit*, *Epcam*, *Cldn1* and *Cd209a*. **(B)** Heatmap of select TLRs, chemokines, cytokines and their receptors for genes differentially expressed between MΦ/DCs, cDC2s, migDCs and pDCs. **(C)** The KEGG pathways enriched in the marker genes of different DCs. **(D)** The ordering of DC populations along pseudotime in a two-dimensional state-space defined by Monocle3 (left) and developmental states of DCs inferred by pseudotime (right). Cell orders are inferred from the expression of the most dispersed genes across DCs. Each point corresponds to a single cell, and each colour represents a DC population. Cells on the same or neighbouring branches are expected to be more hierarchically related. Pie charts show the proportion of cell clusters at the state when multiple clusters are involved. **(E)** Hierarchy clustering of the gene set with similar expression trends in each state of the trajectory. The horizontal axis is the pseudo-time point (the pseudo-time point gradually increases from left to right), the vertical axis is the gene expression level and different colours indicate the level of gene expression level.

Three DC populations were identified, including cDC2, migDC and pDC. The normalised expressions of the top five marker genes revealed exclusive signatures of migDCs and pDCs, implying distinct phenotypic and functional properties of these two clusters (**Figure 4A**). Since MΦ/DCs also showed dendritic cell signatures, such as the high expression of *Thbs1*, *Adam8* and *Ccr2*, it was included in the comparative analysis with the DCs. cDC2s expressed *Tlr1*, *Tlr3*, *Tlr5* and *Tlr6*, while pDCs preferentially expressed *Tlr1*, *Tlr7*, *Tlr9* and *Tlr12*. MΦ/DCs had higher expressions of *Tlr2*, *Tlr4*, *Tlr5*, *Tlr6*, *Tlr8* and *Tlr13* (**Figure 4B**). The expression of many TLRs was absent or reduced in migDCs, except for *Tlr13* in the untreated and control groups. Thus, the cDC2s and pDCs should sense and respond to different innate immune stimuli. Genes encoding chemokines, chemokine receptors and cytokines exhibited distinct expression patterns in the three DCs with different treatments (**Figure 4B**). cDC2 exclusively expressed *Il1r*, *Il18rap* and *Ifngr1*, whereas migDCs had comparatively increased the expressions of *Mmp25*, *Il15*, *Ccl22*, *Ccl17*, *Ccl5*, *Cx3cl1*, *Ccr7*, *Cxcr5* and *Il15ra*. With respect to pDCs, *Ccl25*, *Ccl4* and *Cxcr3* displayed elevation in the control group. In contrast, caerin gel significantly lowered the expression of *Ccl4*. The expression of several other chemokines and receptors was shared between cDC2s and migDCs, including *Cxcl9*, *Cxcl16* and *Ccr6*, which were absent from pDCs. The unique repertoires of chemokines and chemokine receptors expressed by these three DCs indicated divergent functions modulated by the treatments. The expressions of genes playing roles in antigen processing, such as *Fcer1g*, *Ctss* and *Cd86*, were increased more in cDC2 cells. The enrichment analysis of the KEGG pathway identified Th1, Th2 and Th17 cell differentiation, and C-type lectin receptor signalling pathways significantly represented in cDC2s and migDCs (**Figure 4C**).

The trajectory analysis revealed five developmental states in these four DC clusters, where MΦ/DCs emerged at an early pseudotime with a 100% proportion of State-5, as well as on the branch of State-4 (**Figure 4D**). It joined cDC2s (8%), migDCs (1%) and pDCs (0.5%) at State-3, which developed into two states with cDC2 contributed to the entire State-1. The migDCs and pDCs only appeared in a branch at a late developmental stage of DCs represented by similar expression patterns of cytokines/chemokines and their receptors (incl. *Ccl3*, *Tlr4*, *Mmp13*, *CCr5* and *Cxcl2*). The three groups showed cell distributions on the entire trajectory (**Figure 4D**). The caerin gel induced an early appearance of State-1 and 4 and slightly expanded State-2 and 3. The marker genes with similar expression patterns along pseudotime were thus clustered to unravel the function of these states and their function (**Figure 4E**). States-1 and 3 were closely correlated and then hierarchically clustered with State-4, with the high expression of genes related to TNF signalling at late pseudotime, such as *Tnfsf4*, *9*, *10* and *Tnfrsf18*. The genes with antigen-presenting and processing functions were expressed at an early time of State-2 (*Clec4a2*, *Clec4e* and *Clec4d*) and 5 (*Fcgr1* and *Fcgr4*), yet late for State-1 (*Cd86*) (**Supplementary Data 5**).

The caerin group had the highest number of differentially expressed genes in migDCs, compared to the other two groups (**Supplementary Data 2**). The FC and *p*-values of the top 40 significantly regulated genes were summarised in **Figure S6A**. The upregulation of four genes relevant to the activation of NF-κB signalling was detected, including *Bcl3* (43), *Tradd* (44), *Mtdh* (45) and *Ncoa6* (46), indicating more pro-inflammatory migDCs in the caerin group. The overall gene expression of the four DCs was compared, which showed that *Ly6a*, *Tmed7*, *H2-Oa*, *Bag1* and *Cebpb* were significantly elevated by the caerin gel (**Figure S6B**). The pathways more enriched in the DCs of the caerin group included antigen processing and presentation IL-17, CAMs and MAPK signalling pathways (**Figure S6C** and **Supplementary Data 5**).

### Caerin Gel Stimulated the Functions of NK Cells With Different Phenotypes

**Figure 5:**
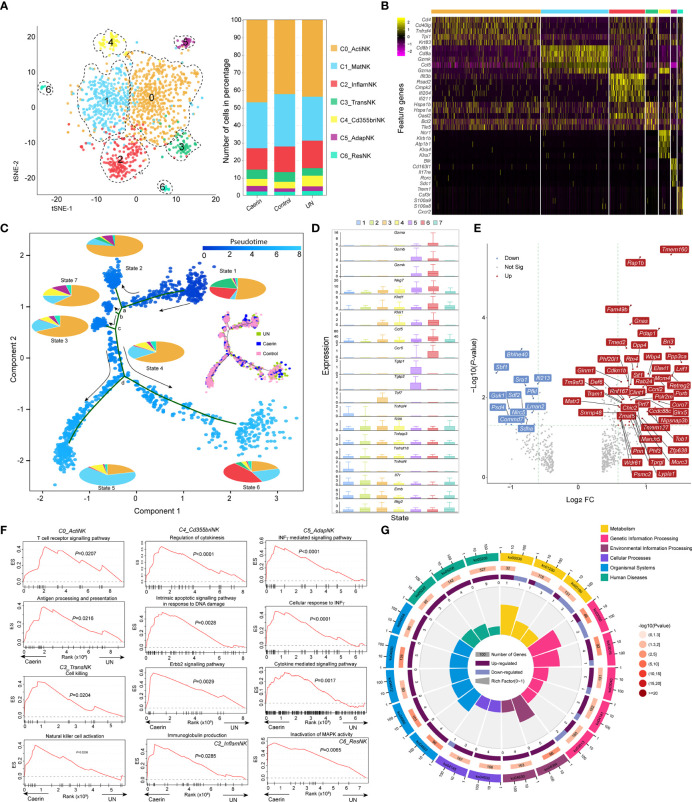
The heterogeneity and function of NK cells were modulated by topical application of caerin 1.1/1.9 gel. **(A)** Two-dimensional t-SNE representation (left) of aligned gene expression data of the subpopulations of NK cells, and the proportions of cell numbers of six NK subpopulations identified in the caerin, control and untreated groups (right). **(B)** Selected enriched genes used for biological identification of each subpopulation (scale: log2 fold change). **(C)** The ordering of NK subpopulations along pseudotime in a two-dimensional state-space defined by Monocle3. Cell orders are inferred from the expression of the most dispersed genes across NK subpopulations. Each point corresponds to a single cell, and each colour in the pie charts represents an NK subpopulation similar to that in **(A)**. The cell distributions on the trajectories in the caerin, control and untreated groups are compared. **(D)** The expression of selected genes associated with functional NK cells is compared throughout seven states identified. **(E)** Volcano graph shows the top 60 genes significantly regulated (FC > 1.5 and *p* < 0.05) in NK cells of the caerin group relative to the untreated group. **(F)** The enrichment of biological processes in different NK subpopulations revealed by GSEA analysis in the caerin group compared to the control group. **(G)** KEGG pathways enriched (*p* < 0.05) in the C0_ActiNK of the caerin group in comparison to the control group (see **Figure S10** for the enrichments in other subpopulations). The classifications and the IDs of KEGG pathways are shown, and the numbers of significantly upregulated and downregulated genes of each KEGG pathways are shown.

The two treatments regulated the expression of genes in NK cells differently compared to the untreated group; the top five maker genes of the caerin group included *Tnfaip3*, *Phf20l1*, *Hnrnpab*, *Cdkn1B* and *Gnas* (**Figure S6A**). A total of seven NK subpopulations (C0 to C6) were identified (**Figure 5A**). The subpopulation C0 and C5 of the caerin group showed higher proportions and were enriched in immune response-relevant pathways, such as T cell receptor signalling, cytokine–cytokine receptor interaction and Th17 cell differentiation (**Figure S6B**). C0 was characterised by the marker genes *Cd4* (47), *Cd40lg* (48) and *Icos* (49) (**Figure 5B**), which represented the phenotype of active NK cells (C0_ActiNK). The C1 displayed significantly higher expressions of *Gzma*, *Gzmk*, *Klrc1* and *Nkg7*, indicating the identity of mature NK cells (C1_MatNK). Many marker genes associated with C2 were involved in interferon induction and activation (*Ifit1*, *Ifit3b*, *Ifit3*, *Isg20*, *Igtp*, *Ifi204* and chemokine encoding gene *Cxcl10*), indicating the stimulation of NK cells by interferons (**Supplementary Data 6**). In addition, C2 appeared to associate with positive regulation of inflammatory responses, as indicated by the high expression of *Cd69* and the enrichment of IFN-γ signalling, toll-like receptor signalling and the pathways related to viral infections. Thus, this subpopulation was referred to as C2_InflamNK.

Many marker genes of C3 appeared relevant to stress response (*Hspa1a*, *Hspa1b*, *Bcl2* and a few mitochondrial enzymes), while few genes appeard directly relevant to the immune functions of NK cells, suggesting the likelihood of a transitional state, which was thus named transitional NK (C3_TransNK) (**Figure 5B** and **Supplementary Data 6**). *Xcl1* and *Sell* were previously identified as the signatures for human CD56^bright^ NK (50); in addition, the exclusive expression of *Ncr1* (i.e., *NKp46*/*Cd335*) to C4 was detected, and thus an identity of CD335^bright^ NK cells could be assigned to the fifth subpopulation (C4_Cd355briNK). *Blk* was only detected as the marker for the C5, and it was found tumour suppressive in chronic myeloid leukaemia stem cells (51) and oncogenic in cutaneous T-cell lymphoma (52). Moreover, the high expressions of *Cd163l1*, *Il17re*, *Rorc* and *Il7r* implied the phenotype of Rorc^+^CD127^+^ NK cells, which exhibited adaptive immune features (53). Thus, this subpopulation was labelled as C5_AdapNK. The C6 exclusively expressed *Trem1*, *Csf3r* and *Cxcr2*, and highly expressed *Alox5ap* and *Ccr1*. This implied that these NK cells were associated with tissue residency (C6_ResNK).

The trajectory analysis showed a developmental progression with several states separated by four branch points (a, b, c and d) (**Figure 5C**). The percentage contribution of each subpopulation within each state was compared, and States-2, 3, 4, and 7 cells were dominated by C0_ActiNK. C5_AdapNK cells were found to mainly develop in States-2, 7 and 3, while its proportion decreased along the pseudotime. Caerin gel caused a more scattered cell distribution at State-1 and a higher State-7 population (**Supplementary File 1**); in addition, there were more cells distributed in the transition from State-4 to State-6 (**Figure 5C**). Developing from branch point a, State-2 showed high expressions of the genes (*Nfkbia*, *Jun*, *Tnfaip3*, *Fos*, *Cxcl2*, *Junb*, *Icos* and *Ltb*) closely associated with immune response, such as the signalling of TNF, T cell receptor and NF-κB (**Figure S7** and **Supplementary Data 6**). Many *Rps* and *Rpl* genes were abundant in the cells of State-7 and State-3 originated from branch points b and c, respectively, suggesting an active state of mRNA processing and translation. Moreover, State-3 cells appeared more proinflammatory, due to the enrichment of TNF-α/NF-κB signalling supported by the upregulation of *Rpl30*, *Rack1*, *Rpl8*, *Rps11* and *Rps13*. Divided by branch point d, State-5 cells had high expressions of *Gzmb*, *Plac8*, *Ly6a*, *Ly6e* and several *Ifit* genes, which were the signatures associated with interferon and cytokine-mediated signalling. In contrast, the elevation of the genes directly signifying functional NK cells, including *Klrd1*, *Klrc1*, *Xcl1*, *Gzmk* and *Nkg7*, was present in State-6.

C1_MatNK, C2_InflamNK and C3_TransNK were dominated by one cell state (**Figure S8A**). In the caerin group, the C1_MatNK and C2_InflamNK cells were visible at higher proportions in State-6 and State-5, respectively, which included more genes related to functional NK cells (**Figure 5D**). There was a substantial proportion of State-3 cells detected in C5_AdapNK of the caerin group, further indicating transcriptome alteration in adaptive immunity. The expression of the top five significantly upregulated genes in each subpopulation of the caerin group showed genes exclusively expressed in C4_Cd355briNK (*Lrrc8c*, *Nol11*, *Asb8* and *Sltm*) and C6_ResNK (*Lmbrd1*, *Gdi1*, *Dusp5*, *Taok3* and *Tm9sf3*), with respect to the untreated group (**Figure S8B**). The NK cell marker *Gzmb* was upregulated significantly in all subpopulations except C1_MatNK of the caerin group (**Figure S8C**).

Several genes associated with neutrophil activation (*Rap1B*, *Bri3*, *Rab24*, *Psmc2* and *Pdap1*) were significantly upregulated in NK cells of the caerin group, while *Nlrc5* and *Commd7*, negatively regulating NF-κB transcription factor activity, were downregulated with respect to the control (**Figure 5E**). *Fam49b* was significantly upregulated in all the subpopulations of the caerin group, and its role as a tumour suppressor in pancreatic ductal adenocarcinoma cells *via* regulating mitochondrial fission was found (54). The upregulation of *Ppp3ca* in most subpopulations suggested a higher-level induction of NFAT signalling (55). The GSEA analysis revealed that gene sets associated with T cell receptor signalling and antigen processing and presentation were enriched in C0_ActiNK of the caerin group, while C3_TransNK was enriched with natural cell activation and cell killing (**Figure 5F**). Moreover, the regulation of cytokinesis and INFγ-mediated signalling pathway was respectively enhanced in C4_Cd355briNK and C5_AdapNK. Inactivation of the MAPK activity pathway was enriched in C6_ResNK. Two interferon stimulation-associated genes (*Bst2* and *Isg20*) were largely expressed in the caerin group, especially in C2_InflamNK. In terms of KEGG pathways, natural killer cell-mediated cytotoxicity, IL-17 signalling pathway and Th1 and Th2 cell differentiation were enriched in C0_ActiNK, with the relevant genes significantly upregulated by caerin gel in comparison to the control (**Figure 5G**). More activated T cell receptor signalling and more pronounced antigen processing and presentation were present in C2_InflamNK and C6_ResNK, respectively (**Figure S9**).

### Caerin Gel Induced More Activated CD8^+^ T Cells Infiltrated to the TME

**Figure 6:**
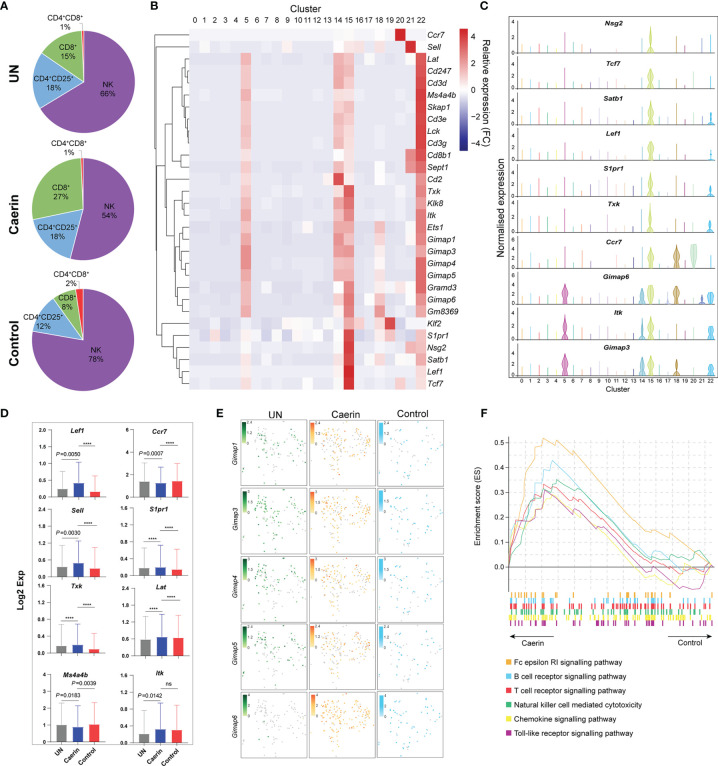
Caerin 1.1/1.9 gel recruited more CD8^+^ T cells with higher activation to TME of TC-1 tumour. **(A)** The proportions of CD4^+^CD8^+^, CD8^+^, CD4^+^CD25^+^ T cells and NK cells detected in the TC-1 tumours untreated, treated with the caerin or control group. **(B)** Hierarchy clustering of the relative expressions of the top 30 marker genes of CD8^+^ T cells (cluster 15) in comparison with other cell populations. **(C)** Violin plots compare the gene expression of selected genes showing significant upregulation (*p* < 0.05) in CD8^+^ T cells with other cell populations, including *Gimap3*, *Itk*, *Gimap6*, *Ccr7*, *Txk*, *S1pr1*, *Lef1*, *Satb1*, *TCF7* and *Nsg2*. **(D)** The expressions (Log_2_ value) of *Lef1*, *Ccr7*, *Sell*, *S1pr1*, *Txk*, *Lat*, *Ms4a4b* and *Itk* in untreated tumours, caerin or control group. A two-tailed Student’s *t*-test is used to evaluate the significance, ^****^ *p* < 0.0001; ns, no significance. **(E)** 2D tSNE plot visualises the cells expressing *Gimap1*, *Gimap3*, *Gimap4*, *Gimap5* and *Gimap6*, in untreated, caerin and control groups, with different normalised gene expression. **(F)** GSEA analysis reveals the enhancement of several immune response relevant KEGG pathways in CD8^+^ T cells of the caerin group with respect to the control group.

In the caerin group, the proportion of CD8^+^ T cells was 2.21%, which was remarkably higher than that of the untreated or control group (**Supplementary Data 1**). CD8^+^ and CD4^+^CD25^+^ T cells were more closely correlated with NK cells than CD4^+^CD8^+^ T cells in a 2D tSNE space (**Figure S10A**). Taking these four cell populations together, CD8^+^ T cells had a much higher proportion of nearly 30% in the caerin group (**Figure 6A**). The expression of selected marker genes of CD8^+^ T cells was also detected mainly in other T cells and NK cells, including those positively regulating the activation of CD8^+^ T cells, such as *Tcf7* (56), *Lef1* (57), *Satb1* (35), *S1pr1* (58) and *Txk* (56) (**Figure 6B**). The expressions of *Nsg2*, *Tcf7*, *Satb1*, *Lef1* and *S1pr1* were almost exclusively and highly expressed in CD8^+^ T cells (**Figure 6C**). *Ccr7* showed a higher expression in B cells and migDCs, while *Itk* exhibited an elevation in NK and B cells. The expression (in Log_2_ value) of nine genes related to the regulation of T cell activation was statistically compared among the three groups (**Figure 6D**). *Lef1*, *Sell*, *Txk*, *Tpt1*, *Lat*, *Itk* and *S1pr1* were upregulated, whereas *Ccr7* and *Ms4a4b* were downregulated. Several *Gimap* genes, previously shown to be involved in lymphocyte development, or associated with inflammatory and autoimmune diseases (59), were detected as the marker genes for CD8^+^ T cells, including *Gimap1*, *3*, *4*, *5* and *6* (**Figure 6E**). It showed that more cells expressed higher levels of these *Gimap* genes, especially Gimap6, in the caerin group. The GSEA analysis revealed that the signalling of B cells, T cells, chemokine and toll-like receptor as well as natural killer cell-mediated cytotoxicity KEGG pathways were enriched in the caerin group with respect to the control group (**Figure 6F**). The Reactome pathways enriched by the unique marker genes of CD8^+^ T cells of the caerin group included signalling of several FGFR mutant-related pathways, interleukins and cytokines in the immune system (**Figure S10B**). This further suggested a potentially more activated state of CD8^+^ T cells induced by the caerin gel treatment.

### Higher Immune Response due to the Topical Application of Caerin Gel Revealed by TMT10plex Labelling Quantitative Proteomics

**Figure 7:**
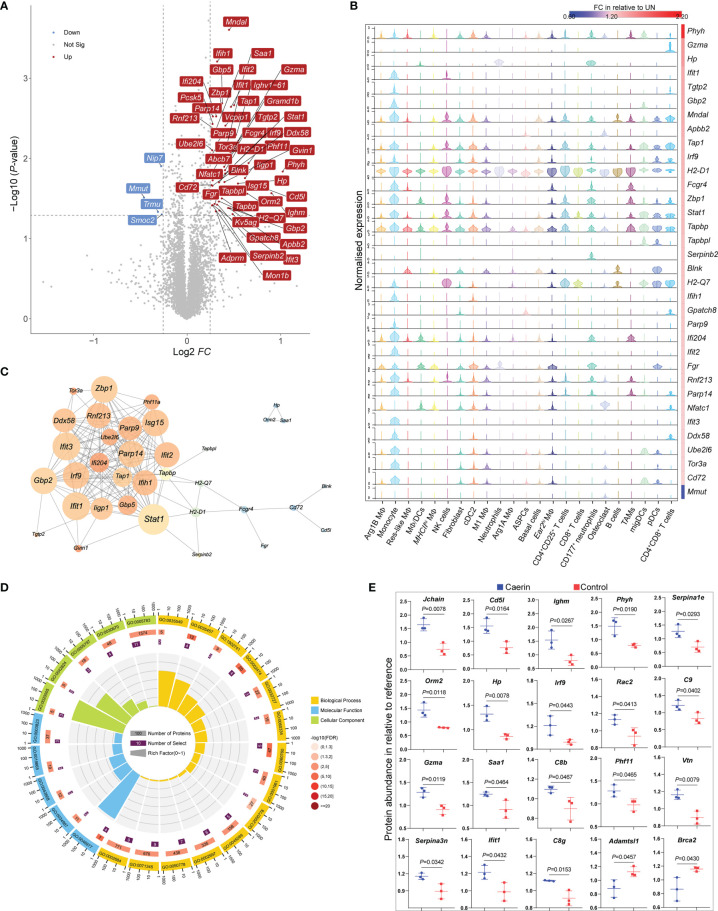
TMT10plex-labelling quantitative proteomic analysis of TC-1 tumour in the untreated, caerin or control gel groups. **(A)** The volcano graph shows proteins significantly regulated (FC > 1.5, *p* < 0.05) only in the caerin group with respect to the untreated group. **(B)** The correlation between the normalised gene expression (determined by scRNA-seq analysis) of the proteins showing significant upregulation only in the caerin group, and the fold change of these proteins relative to the untreated group. **(C)** PPIs of significantly upregulated proteins in the caerin group. The size of the node corresponds to the degree of interaction, and the colour indicates the number of neighbour(s) (see **Figure S13** for the statistical analysis of the PPI). **(D)** Gene ontology enrichment analysis of the PPI network shown in **(C)** (See **Supplementary Data 8** for more details). **(E)** Comparison of the abundance of selected proteins significantly regulated (*p* < 0.05) in the untreated and caerin groups relative to reference. A two-tailed Student’s *t*-test was used to evaluate the significance.

The quantitative proteomic analysis showed that more proteins were significantly regulated by the caerin gel, with a total of 48 proteins upregulated uniquely, including those related to immune response (including Stat1, Gzma, Ifit1, Ifit3, Lfih1, Tap1 and Cd72) (**Figure 7A**; **Supplementary Data 7** and **Figure S11**). Several upregulated proteins were exclusively correlated with the genes (*Ifih1*, *Tgtp2*, *Gbp2* and *Ifit3*) highly expressed in monocytes identified by the scRNA-seq analysis (**Figure 7B**). The high quantities of several proteins (H2-Q7, Tap1, Tapbpl, Gzma and Stat1) were closely correlated with their gene expression in T cells, DCs and NK cells. The exclusive expression of Hp and Serpinb2 to neutrophils was detected. The gene expression of H2-D1 was significantly upregulated in all cells, as well as the diverse distribution of Tapbp, Stat1 and Zbp1, implying that they were largely regulated by the caerin gel.

The protein–protein interaction (PPI) analysis of upregulated proteins identified intensive interactions only present in the caerin group (**Figure 7C** and **Figure S13**). *Stat1* was found as the node with the highest degree, while it was a marker gene for monocytes, NK cells (also C2_InflamNK), CD4^+^CD25^+^ T cells and migDCs. The gene ontology enrichment analysis of this PPI network revealed biological processes related to the regulation of immune response, such as defence response (FDR = 1.52E-13), immune system process (FDR = 5.12E-13) and innate immune response (FDR = 6.72E-12) (**Supplementary Data 8**). More specifically, the positive regulations of INFα/β secretion (supported by Gbp5, Iigp1, Stat1, Ifit1, Ifit3, Ifi204 and Gbp2), response to cytokine stimulus (Ifih1, Zbp1, Parp14, Ddx58 and Parp9) and antigen processing and presentation of peptide antigen *via* MHC class I (Tapbp, H2-Q7, Tap1, and H2-D1) were largely enhanced by the caerin gel (**Figure 7D**). In terms of molecular function, the binding of TAP, immunoglobulin/receptor and T cell receptor and antigen processing and presentation were enriched in the caerin group relative to the other two groups.

The FC values of proteins differentially regulated by caerin and control were compared (**Figure 7E**); many upregulated proteins (such as C9, C8b, C8g, Igj, vtn and Serpina3n) appeared to play roles in the processes related to immune response and complement activation. This was in accordance with the identification of more immune activity of MΦs and DCs, as well as the recruitment of activated CD8^+^ T cells in the TME of the caerin group, as suggested by scRNA-seq. The KEGG pathways enriched in upregulated proteins were thus highly associated with signalling in immune response and process (**Supplementary Data 8**), such as antigen processing and presentation (FDR = 6.0E-4), the signalling of NOD-like receptor (FDR=6.0E-4) and natural killer cell-mediated cytotoxicity (FDR = 7.7E-3).

## Discussion

The immunoregulatory properties of some host defence Anura peptides have been documented, such as inhibiting production of IL-10 and transforming growth factor-β (TGF-β) from unstimulated and ConA-stimulated PBM cells (60) and stimulating production of the pro-inflammatory cytokines (incl. TNF-α, IL-1β and IL-12) (61). Caerin 1.1 and 1.9 were able to inhibit multiple types of tumour growth *in vitro*, resulting in the apoptosis of the tumour cells. They also inhibited TC-1 tumour growth *in vivo* when locally injected to tumour, and the tumour inhibition effect is dependent on the existence of intact adaptive immune systems, as their tumour inhibition effect disappeared in Rag^-/-^ mice, which lacks B and T cells (62) and mice depleted of T cells (unpublished data). The anti-proliferative activity of caerin 1.1 and 1.9 against HeLa cells *in vitro* was investigated, which found that the TNF-α-dependent apoptosis signals were stimulated by the caerin peptides (22). In a recent study, we have shown that intratumoral injection of the caerin 1.1/1.9 mixture significantly prolonged the survival time of TC-1 tumour-bearing mice that were immunised with an HPV16 E7 peptide-based vaccine along with IL-10 and PD-1 blockade (23). The TME was largely altered to a higher immune activation level, possibly with *Stat1* as a key modulator, which synergistically functions with the activated NF-κB pathway to induce more iNOS and triggers the recruitment of T cells. Caerin 1.1 and 1.9 in gel form were able to inhibit TC-1 growth when topically applied to subcutaneously transplanted TC-1 tumour, while the pure gel matrix did not reduce the tumour mass, compared to untreated mice (21), which meant the tumour suppression of the caerin gel was due to the activity of caerin 1.1 and 1.9.

In this study, the scRNA-seq analysis uncovered that the topical application of the caerin gel altered the cell heterogeneity and function of tumour infiltrating leukocytes of the TME, especially macrophages, DCs and CD8^+^ T cells. The proportions of active and adaptive NK cells were elevated with the treatment of the caerin gel; additionally, the adaptive NK cells exhibited a more pro-inflammatory phenotype, and the Cd355^bright^ NK cells showed high levels of interferon and cytokine-mediated signalling. These effects modulated the TME to become more pro-inflammatory, which may favour tumour rejection; this was in accordance with the quantitative proteomic analysis. This might be due to the intensive interaction between the caerin peptides, the tumour and the TME, as a relatively high penetration magnitude of caerin 1.9 through the epidermis of mice was clearly observed.

The INFα response was largely activated in the four *Arg1^hi^* MΦs of the caerin group. Moreover, the INFγ response in the Arg2B and Arg2A MΦs was enhanced, which meant that a proinflammatory TME was formed in the caerin group, with potent tumour growth inhibitory effects (63) and elevated immunosurveillance. This may be associated with the significant upregulation of *Cebpb* by the caerin gel, since IFNα was found to increase the expression of *Cebpb via* recruiting *Stat1* and *Stat5* (64). Notably, the elevation of *Stat1* was confirmed by the proteomic analysis. *Cebpb* is a member of the CCAAT/Enhancer Binding Protein (C/EBP) family of transcription factors, which are activated by IL-17 (65–68). *Cebpb* binds to the *Il23r* promoter in Th17 cells and bone marrow-derived myeloid cells (69) and regulates the Fcγ receptor-mediated induction of *TNFα*, *Cxcl2* and *Ccl3* in macrophages (70).

Dendritic cells are professional antigen-presenting cells that link the innate and adaptive arms of the immune system. The caerin gel produced greater modulation in the overall function of three DCs, as reflected by the significant enrichment of antigen processing and presentation and CAMs. The caerin gel treatment upregulated *H2-Oa*, a gene facilitating peptide loading of MHC class II molecules (71) *via* interacting with *Irf4* (72). *Irf4* plays a pivotal role in the development and function of several autoimmune-associated cells, including DCs (73). In the migDCs of the caerin group, both *Tradd* and Cd48 were significantly upregulated; the nuclear form of *Tradd* was found as a tumour suppressor by preventing ubiquitination and degradation of isoform p19ARF/ARF of *Cdkn2a* by *Trip12* (74), while the elevation of *Cd48* expression correlated with the activation of CD4^+^ T cells (75). The pDCs expressed higher levels of transcripts associated with tissue repair such as the metalloproteinase *Mmp9*, which was found to modulate cytokine activity through the activation of TGF-β (76) and the inhibition of *Il23* expression (77). Furthermore, the elevated expression of *Ly6a* was detected, which correlates with the activation of *Tlr7* and *Tlr9* in pDCs (78), triggering signalling cascades associated with a proinflammatory cytokine response (79). These observations suggested that the proinflammatory DC phenotype was induced by the caerin gel.

Caerin gel treatment significantly upregulated the expression of *Tnfaip3* in NK cells. *Tnfaip3* regulates TCR/CD28-mediated NF-κB activation and TCR mediated survival (80), as well as necroptosis and IFNγ release (81). *Il7r* was highly expressed in C0_ActiNK and C5_AdapNK of the caerin group, and *Il7r*/*Il7* signalling increases cytokine production and elevates the cytotoxicity and survival of CD56^bright^ NK cells (82). The higher proportions of more immune active State-5/6 cells in Cd355^bright^ and resident-like NK cells indicated the activation of NK function, while State-1/3 cells implied an active proliferative state of adaptive NK cells.

The CD8^+^ T cell population was remarkably expanded in the caerin group, which accorded with a recent study showing that the caerin gel increased the population of CD45^+^CD3^+^ T cells (21). The expressions of *Lef1*, *S1pr1*, *Txk*, *Sell*, *Itk* and *Lat* were significantly upregulated by the caerin gel. *Lef1* has been known to be critical for the production of T cells (83). The critical roles of *Lat* in the activation of T cells (84, 85) and the cytotoxicity of CD8^+^ T cells (86) were demonstrated. The signalling of *S1pr1*/*S1p* plays critical roles in the activation and subset polarisation of T lymphocyte (87–89), and the regulation of effector CD8^+^ T cells that egress from the draining lymph node (dLN) by *S1pr1* expression was identified (58). *Ccr7* plays a critical role in the localisation and retention of T cells within the LN paracortex (90, 91); particularly, its expression greatly contributes to the homing of memory CD8^+^ T cells into the LNs, liver, lung and bone marrow (92). The activated effector CD8^+^ T cells downregulates *Ccr7* to egress from the reactive LN into the circulation (58, 93). *Ms4a4b* is expressed in naïve CD8^+^ T cells in thymocytes at pre-commitment and mature developmental stages (94) and contributes negative feedback to T cell activation in general (95). The significant upregulation of *S1pr1* and downregulation of *Ccr7* and *Ms4a4b* imply that more effector CD8^+^ T cells were released by LN to the TME. *Gimap* members play vital roles in T cells (82, 96, 97). *Gimap5* is important especially for the survival of the CD8^+^ lineage and mature peripheral T cells (98, 99). The expression of *Gimap1* starts in hematopoietic precursors critical to early T cell development (59). The requirement for *Gimap6* in the maintenance of T cells towards developing a normal peripheral adaptive immune system was demonstrated (100). Thus, the elevated expression of *Gimap* genes strongly suggested that more activated CD8^+^ T cells were induced by the caerin gel. *Txk* and *Itk* belong to the Tec family tyrosine kinase with the functions in T cell activation and mature T-cell differentiation (56, 101). The association of *Sell* with the effector-to-memory transition of CD8^+^ T cells was characterised (102). Thus, the regulation of these genes might work synergistically to result in more activated CD8^+^ T cells in the TME of the caerin group, especially effector and memory transition of CD8^+^ T cells.

The quantitative proteomics revealed that the caerin gel induced a higher immune response in the TME, associated with the elevation of Gzma, Ifit1, Tgtp2, Tap1, Irf9 and Stat1. Stat1 plays a key role in mediating responses to all interferon types, displaying anti-tumour effects on several cancers (103). It functions *via* the interaction with Irf9 to activate the interferon-stimulated genes in the nucleus, thereby enhancing the cellular immunity (104), while the elevation of Irf9 content was exclusively detected in the caerin group. Phyh (peroxisomal phytanoyl-CoA dioxygenase) regulates peroxisomal fatty acid β-oxidation metabolism and ROS conversion (105); its concentration positively correlates with potential tumour suppressive environment (106, 107), working synergistically with *iNOS* induced by *Stat1*, which may trigger the recruitment of CTLs (108, 109). This appeared consistent with more activated CD8^+^ T cells detected by scRNA-seq. Jchain was the top protein significantly upregulated by caerin gel with respect to the control. It was found to emerge early in the evolution of the immune system and predicted to play roles in the dimerisation and transepithelial transportation of IgA (110). Its roles in B cell differentiation and activation (111), as well as intrathymic stages of T cell differentiation, have been documented (112). This means that the caerin peptides might stimulate the signals for T cell production in thymus *via* the elevation of Jchain, and consequently more T cells egress from LN as reflected by the scRNA-seq analysis. A proteomic study on hepatocellular carcinoma identified a high expression of Jchain, together with Cd5l and Lgals3bp, which positively correlated with the response of the chemotherapeutic agent sorafenib (113); their downregulation during tumorigenesis was possibly due to the immunosuppressive effects of the tumour cells. In addition, Jchain is upregulated significantly at the protein level in normal lung tissue adjacent to the tumour, indicating its role in responding to tumour cells and/or the TME (114). Both Jchain and Cd5l were more abundant in the caerin group relative to the control, implying a more tumour-suppressive TME.

In conclusion, the topical application of caerin 1.1/1.9 gel expanded immune-activating macrophages, activated innate immune response in NK cells and DCs and significantly induced more activated CD8^+^ T cells. The developmental process of NK cells was altered with immune response enhanced in adaptive NK cells. It appeared that the two caerin peptides acted as immunomodulators acting through non-linear signalling pathways of the immune system in the TME. This complexity became evident by examining the PPI network of the proteins significantly upregulated in the caerin group, and several of those proteins were key modulators on different pathways. These proteins in turn interacted with many more secondary effectors, which was consistent with the scRNA-seq observations that the expression of hundreds of genes changes when tumours were treated topically by the caerin gel. Harnessing the significantly regulated genes and proteins preferentially enriched in the immune active cell populations may provide a valuable resource for researchers in the field.

## Materials and Methods

### Mice

Six-to-eight-week-old, specific pathogen-free adult female C57BL/6 (H-2b) mice were ordered from the Animal Resource Centre of Guangdong Province and kept at the Animal Facility of the First Affiliated Hospital of Guangdong Pharmaceutical University. Experiments were approved by Animal Experimentation Ethics Committee (Ethics Approval Number: FAHGPU20160316). Five mice were kept in each cage on a 12-h light/darkness cycle (22°C and 75% humidity), provided with sterilised standard mouse food and water. TC-1 tumour-bearing mice were *i.p.* injected with 1% sodium pentobarbital. Mice were euthanised by CO_2_ inhalation at the end of the experiment.

### Cell Line, Peptide Synthesis and Gel Preparation

A murine TC-1 cell line transformed with HPV16 E6/E7 was obtained from Shanghai Institute for Cell Resources Centre and cultured following the protocol described elsewhere (21). Caerin 1.1 (GLLSVLGSVAKHVLPHVVPVIAEHL-NH_2_), caerin 1.9 (GLFGVLGSIAKHVLPHVVPVIAEKL-NH_2_) and the control peptide P3 (GTELPSPPSVWFEAEFK-OH), were synthesised (purity>99%) (Mimotopes Proprietary Limited, Wuxi, China). The lipopolysaccharide concentrations of caerin 1.1, caerin 1.9 and P3 were 0.03, 0.03 and 0.44 EU/ml respectively, as measured by Kinetic Turbidimetric Assay (Xiamen Bioendo Technology Co., Ltd).

Poloxamer 407 (WPAK592B) and poloxamer 188 (WPAK539B) were purchased from Badische Anilin-und-Soda-Fabrik (Ludwigshafen, Germany). The gel was prepared as previously described (21). Briefly, 46 g of poloxamer 407 and 10 g of poloxamer 188 were dissolved in 200 ml of distilled water, and caerin 1.1 and caerin 1.9 were then added. The solution was mixed thoroughly and filtered through a 0.22-μm membrane filter to prepare a 20-mg/ml gel and stored at 4°C.

### Tumour Challenge and Topical Application of the Gels

TC-1 cells, at approximately 70% confluency, were harvested with 0.25% trypsin-EDTA solution and washed with PBS. 5 × 10^5^ cells/mouse in 0.2 ml of PBS were injected subcutaneously into the left flank. TC-1 tumour-bearing mice were either treated with caerin gel or control gel or left untreated for 7 consecutive days by applying 20 μl of each gel onto the shaved skin surface above the tumour. Two days after the final treatment, mice were sacrificed, and the tumours were isolated and weighed.

### Confocal Microscopy

Six-week-old female C57BL/6 mice were anesthetised by intraperitoneal injection of pentobarbital before hairs on the dorsal side of the ear were shaved and gently wiped with normal saline and dried naturally. The mice were treated with thermosensitive gels containing FITC-labelled caerin 1.9 or P3, by evenly applying 100 μl of the gels to the shaved areas for 5 min. The ear skin surface was then gently rinsed with distilled water three times to remove any residual gels and then cut, and sections of 6-μm thickness were stained with DAPI. The samples were observed using 405 and 488 nm by a Zeiss LSM 880 Airyscan confocal microscope (Zeiss, Germany). Dual-channel combined imaging videos were recorded.

### Isolation of Tumour-Infiltrating CD45^+^ Cells and Single-Cell Transcriptome

TC-1 tumours were cut into 2 × 2 mm pieces; digested in 2.35 ml of RPMI 1640, 100 µl of Enzyme D, 50 µl of Enzyme R and 12.5 µl of Enzyme A into a gentleMACS C Tube; and disassociated using gentle MACS Dissociator from Miltenyi (Gladbach, Germany). After the removal of dead cells and cell debris, the remaining cells were labelled with CD45 microbeads (130–110–618). The viability of the CD45^+^ cells were more than 80% of total cells confirmed by flow cytometry and trypan blue staining. Cells were washed once with ice-cold PBS containing 10% foetal bovine serum post sorting and counted using a hemocytometer. After that, the cells were loaded to a 10x chromium machine (10x Genomics, San Francisco, CA) and run through the library preparation procedures following guidance from the Chromium Single Cell 3′ Reagent Kits v2 (more details were provided in the **Supplementary Methods**).

### Protein Extraction and Quantitative Proteomic Analysis

The tumour samples were the same biological triplicates from which the CD45^+^ cells were extracted for scRNA-seq. Certain amounts of samples containing 500 µg of protein were subjected to trypsin digestion by the filter-aided proteome preparation (FASP) described elsewhere (115) followed by LC-MS/MS analysis using a Q Exactive hybrid quadrupole-orbitrap mass spectrometer (Thermo Fisher Scientific, Waltham, MA, USA) (see **Supplementary Materials** for detailed method).

### PPI Analysis

Interactions among significantly regulated proteins were predicted using STRING (116). A required interaction score of 0.700 was selected for all PPI, to highlight the most confident interactions. Neither the first nor second shell of the PPI was included. Protein without any interaction was excluded.

### Gene Ontology, Pathway and GSEA Analysis

The gene ontology terms, including biological process, molecular function and cellular component, were annotated using STRING, and the enrichment of was analysed accordingly. The enrichment of KEGG pathways (117) and Reactome pathways (118) was assessed based on the significantly upregulated genes (*p* < 0.05) of different cell populations/subpopulations. The genes differentially expressed in three groups were analysed by Gene Set Enrichment Analysis (GSEA) with *p* < 0.05 using GSEA v4.1.0 (119).

## Data Availability Statement

The datasets presented in this study can be found in online repositories. The names of the repository/repositories and accession number(s) can be found in the following: https://singlecell.broadinstitute.org/single_cell, SCP1371; http://www.proteomexchange.org/, PXD025779.

## Ethics Statement

The animal study was reviewed and approved by the Animal Experimentation Ethics Committee of the First Affiliated Hospital of Guangdong Pharmaceutical University (Ethics Approval Number: FAHGPU20160316).

## Author Contributions

Conceptualisation and design, TW and XL. Experimental work, GN, YL, PPZ, SC, and XW. Data process, curation and visualisation, GN, HL, TW, PZ, and XL. Analysis and interpretation, TW, PZ, and XL. Writing-original draft preparation, GN, TW, and XL. Writing-review and editing, HL, CF, TW, PZ, XL, MW, and GC. Project administration: XW and GC. All authors contributed to the article and approved the submitted version.

## Funding

This study was supported by the Deng Feng project of Foshan First People’s Hospital (2019A008), Foshan Municipal Government (2015AG1003), Guangdong Provincial Government (2016A020213001) of China, National Science Foundation of China (31971355), and Genecology MCR Seed Funding of University of the Sunshine Coast. The funders were not involved in the design, data collection and analysis, preparation or publication of the manuscript.

## Conflict of Interest

The authors declare that the research was conducted in the absence of any commercial or financial relationships that could be construed as a potential conflict of interest.

## Publisher’s Note

All claims expressed in this article are solely those of the authors and do not necessarily represent those of their affiliated organizations, or those of the publisher, the editors and the reviewers. Any product that may be evaluated in this article, or claim that may be made by its manufacturer, is not guaranteed or endorsed by the publisher.
