# Supplementary material for: Topical Application of Temperature-Sensitive Gel Containing Caerin 1.1 and 1.9 Peptides on TC-1 Tumour-Bearing Mice Induced High-Level Immune Response in the Tumour Microenvironment
Source: Front Oncol. 2021 Nov 11;11:754770. doi: 10.3389/fonc.2021.754770 (PMC8632150; doi:10.3389/fonc.2021.754770)
Supplement: Supplementary file 1 [file DataSheet_1.docx]

Supplementary Material

# Supplementary Data

Datasets of single cell RNA sequencing related to this article can be found at (https://singlecell.broadinstitute.org/single_cell, the accession number SCP1371), hosted at the Institute Single Cell Portal. Datasets of quantitative proteomics can be found at (http://www.ebi.ac.uk/pride, the accession number identifier PXD025779), hosted at the ProteomeXchange Consortium via the PRIDE partner repository.

**Supplementary Data 1** Statistical analysis and quality control of scRNA-seq results, and the cell numbers of different clusters in three groups.

**Supplementary Data 2** The normalised expression of top 5 marker genes of each cell cluster; all significantly upregulated genes of each cell cluster; the gene expression of the marker genes in all clusters. The annotations of these genes are shown.

**Supplementary Data 3** The top 20 enriched biological processes in all MΦ populations.

**Supplementary Data 4** The genes differentially expressed with significance (*P*<0.05) in Arg1B MΦ, MΦ/DC, Arg1A and *Ear2^hi^* MΦ populations between the caerin and control, or the caerin and untreated groups.

**Supplementary Data 5** The genes (incl. the annotations) differentially expressed on two branches of the NK cell trajectories.

**Supplementary Data 6** The subpopulation analysis of NK cells, including the marker genes of each subpopulation and each state derived from the cell developmental trajectory, the genes differentially expressed in the caerin group with respect to the control group, and the enrichment analysis of KEGG pathways.

**Supplementary Data 7** TMT10plex-labelling proteomic analysis results, including quantifiable proteins (FC > 1.2 and *P* < 0.05) highlighted with red (upregulated) and green (downregulated) colours, all identified proteins and supporting peptides.

**Supplementary Data 8** Gene ontology and pathway (KEGG and Reactome) enrichment analysis of the PPIs shown in **Figure 7C**.

**Supplementary Video 1** Confocal microscope scanning of the section of mouse ear skin treated by FITC-labelled control peptide gel at 5min.

**Supplementary Video 2** Confocal microscope scanning of the section of mouse ear skin treated by FITC-labelled caerin 1.9 gel at 5min.

# Supplementary Figures


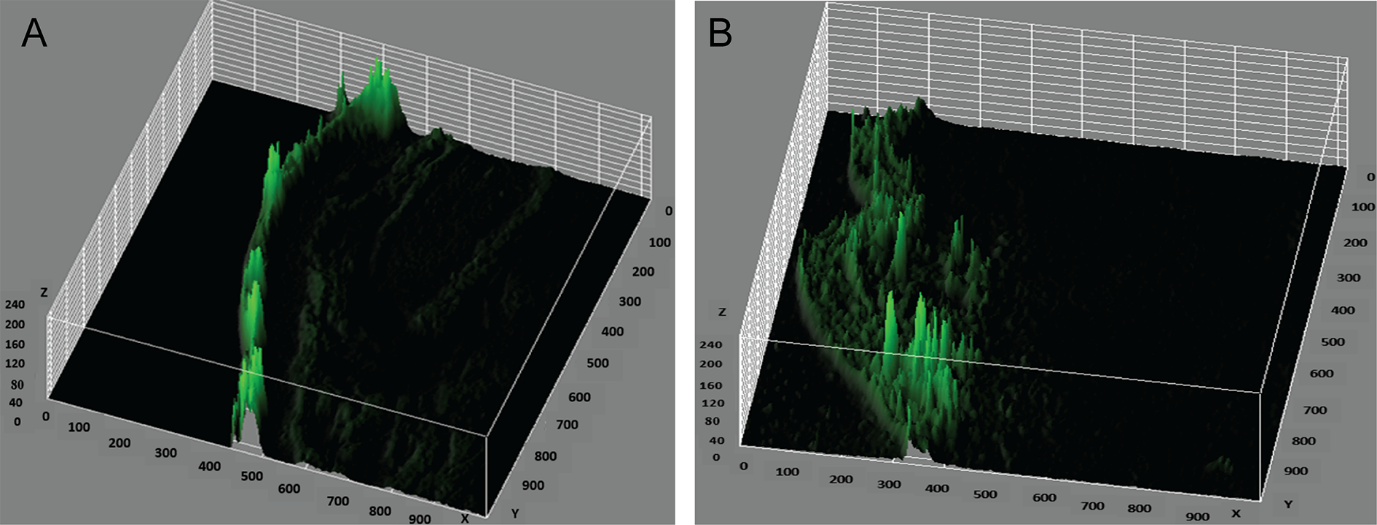


**Figure S1** The distribution of green fluorescence (FITC-labelled P3 or caerin 1.9) in the sections of ear skin treated with the control (A) and caerin (B) gels at 5mins, associated with **Figure 1B** and **1C**.


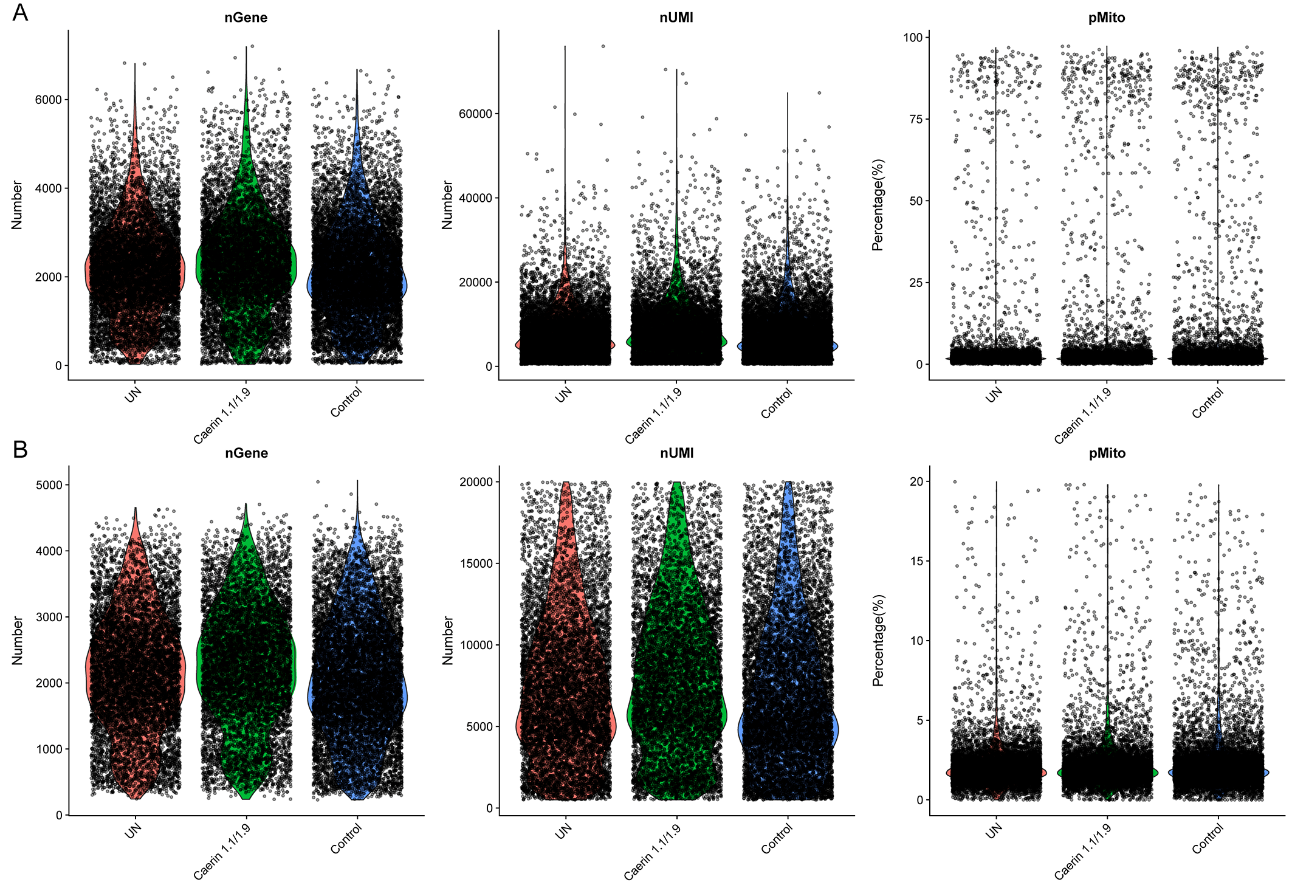


**Figure S2** Basic information of cells in each sample before (A) and after (B) scRNAseq data filtration. Left: distribution of the number of genes detected in individual cells of each sample (Y axis); middle: distribution of the total number of UMIs detected in individual cells of each sample (Y axis); right: distribution of mitochondrial gene expression in individual cells of each sample The percentage of the amount (Y axis) distribution (Note: The cells in apoptosis usually have abnormally high mitochondrial gene expression, and the mitochondrial gene expression level can be used as a reference indicator for identifying low-quality cells in the sample).


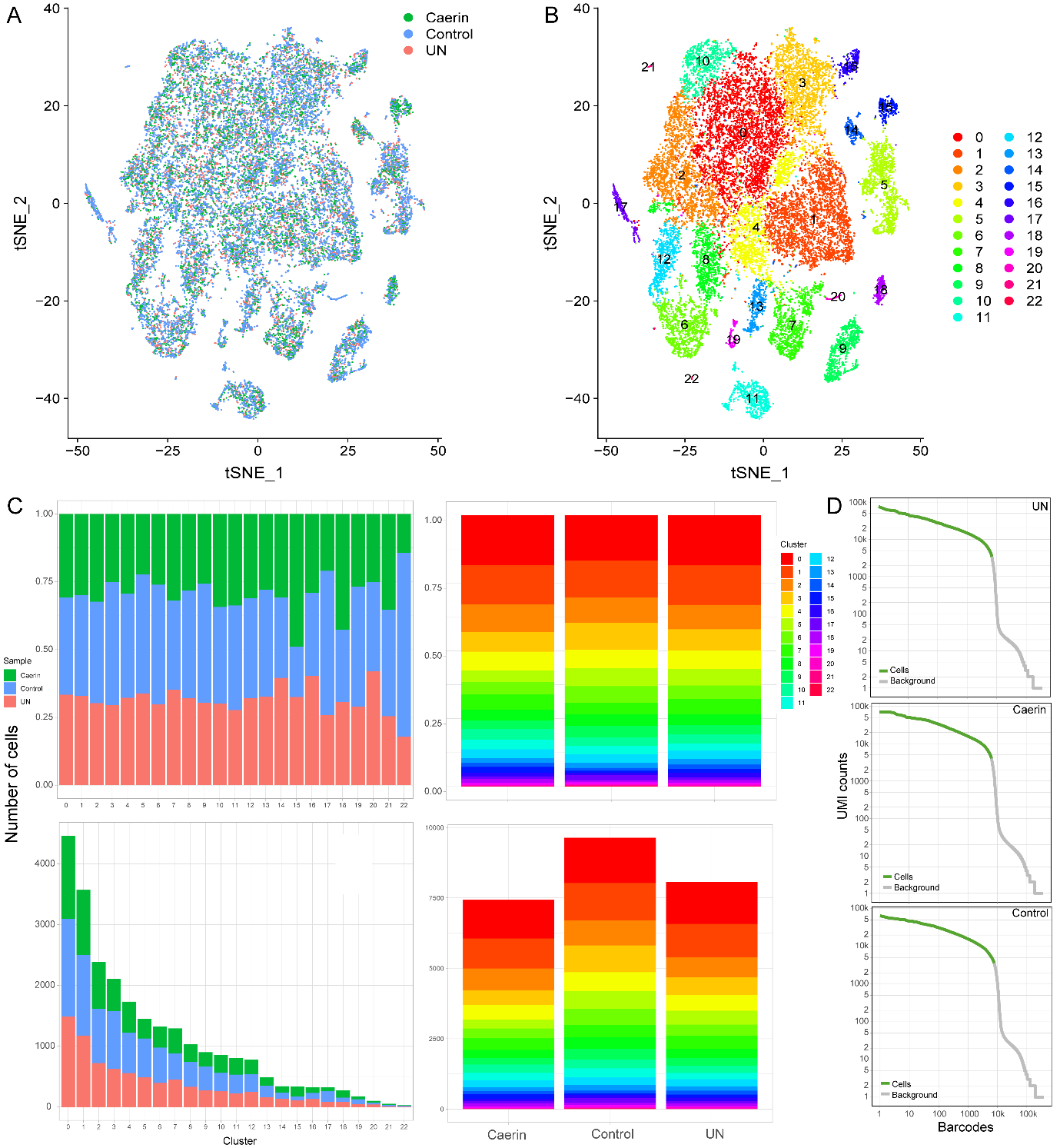


**Figure S3** The 2-dimensional tSNE graph of single cell population distribution in in the untreated, caerin and control groups (A) and cell population classification (B). (C) The stacked graph of the number of cells and the percentage of cells in each cluster in untreated and treated samples. (D) Cell validation graph, colour codes, green-the barcodes of valid cells, grey-background noise.


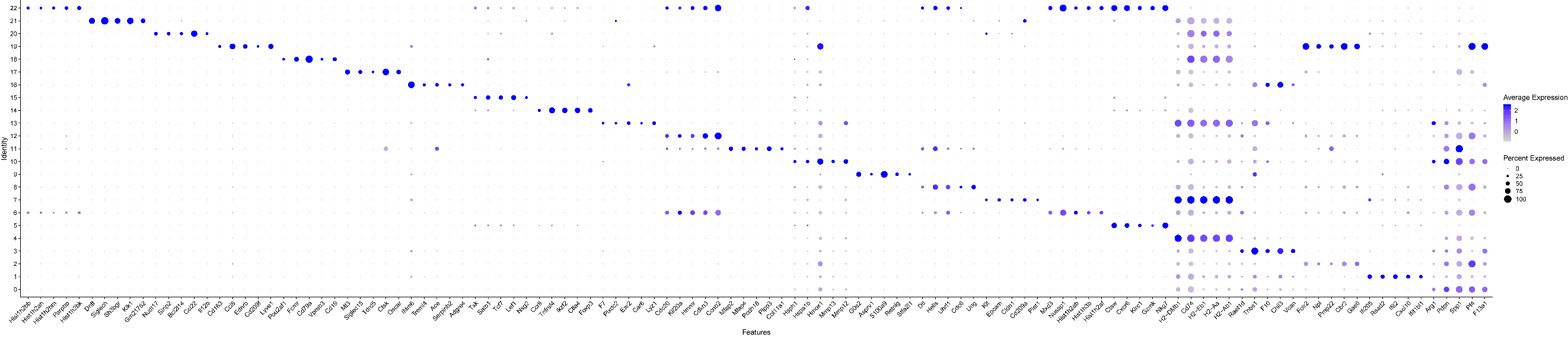


**Figure S4** Bubble map compares the average and the percentage expression of the top 5 marker genes in different cell populations identified by the scRNA-seq analysis of the CD45^+^ cells isolated from the TC-1 tumours.


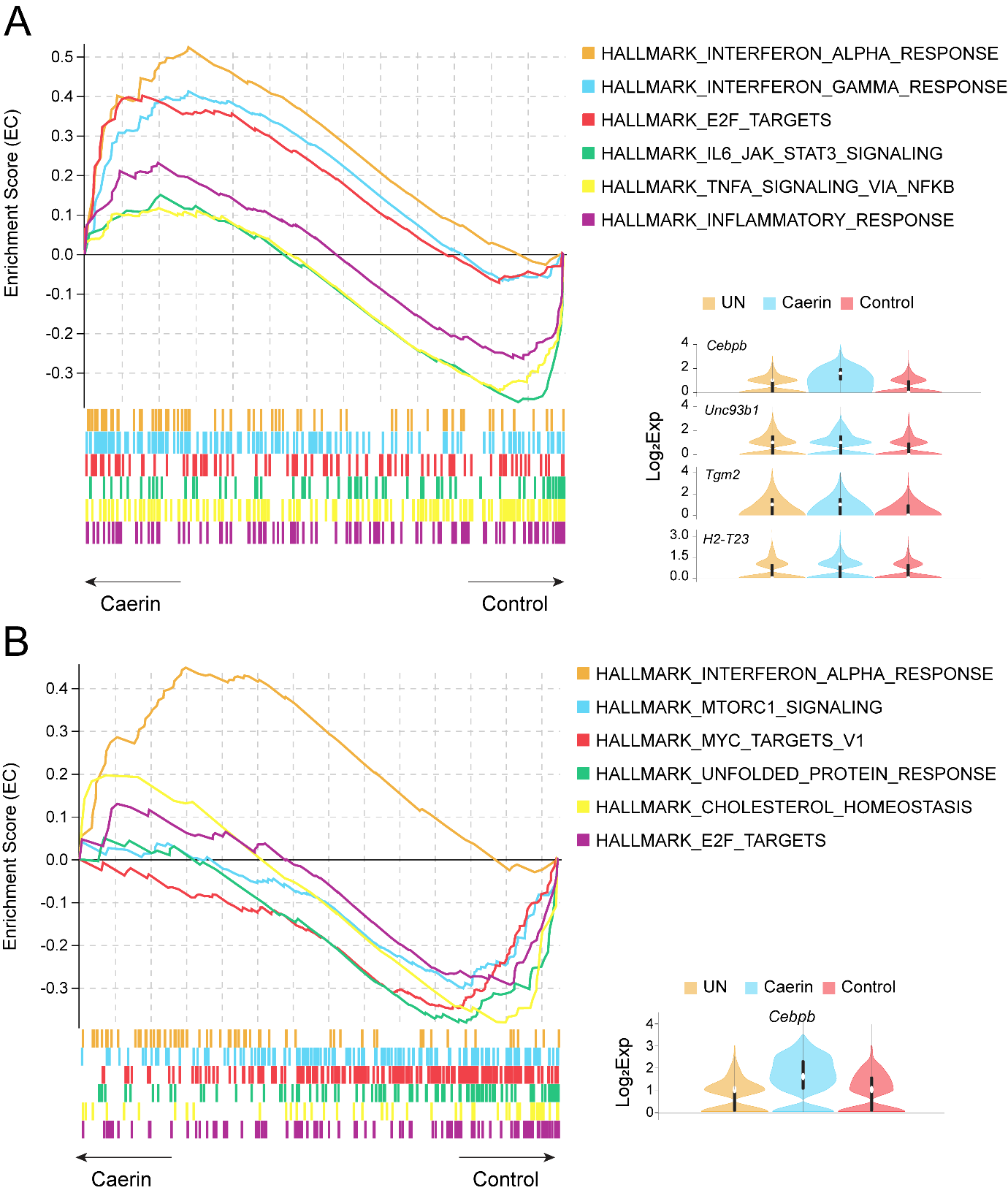


**Figure S5** The GSEA analysis of Hallmark pathways enriched in MΦ/DCs (A) and *Ear2^hi^* MΦs (B) of the caerin group in comparison to the control group. The expression (Log_2_ value) of the genes upregulated significantly in MΦ/DCs or *Ear2^hi^* MΦs of the caerin group with respect to both the untreated and the control group were displayed, respectively.


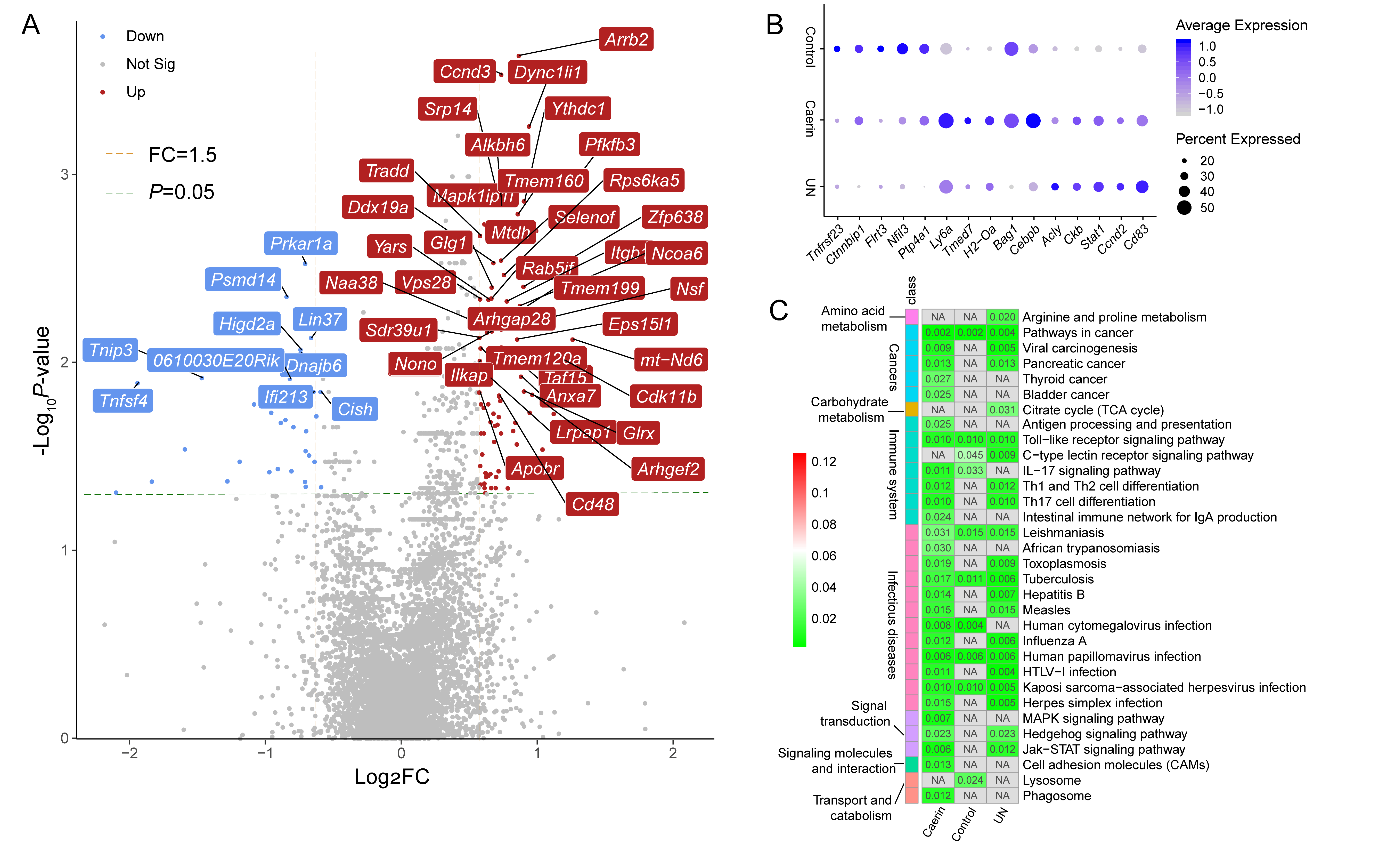


**Figure S6** Caerin gel induced more proinflammatory DCs in the TME. (A) The volcano graph shows maker genes significantly regulated (FC > 1.5, *P* < 0.05) only in the caerin 1.1/1.9 group with respect to the untreated group. (B) Bubble map of the average expression of top genes differentiating DCs of three groups, including *Tnfrsf23*, *Ctnnbip1*, *Flrt3*, *Nfil3*, *Ptp4a1*, *Cd83*, *Ccnd2*, *Stat1*, *Ckb*, *Acly*, *Cebpb*, *Bag1*, *H2*-*Oa*, *Tmed7* and *Ly6a.* (C) Random forest analysis compares KEGG pathways in eight categories in DCs of different groups.


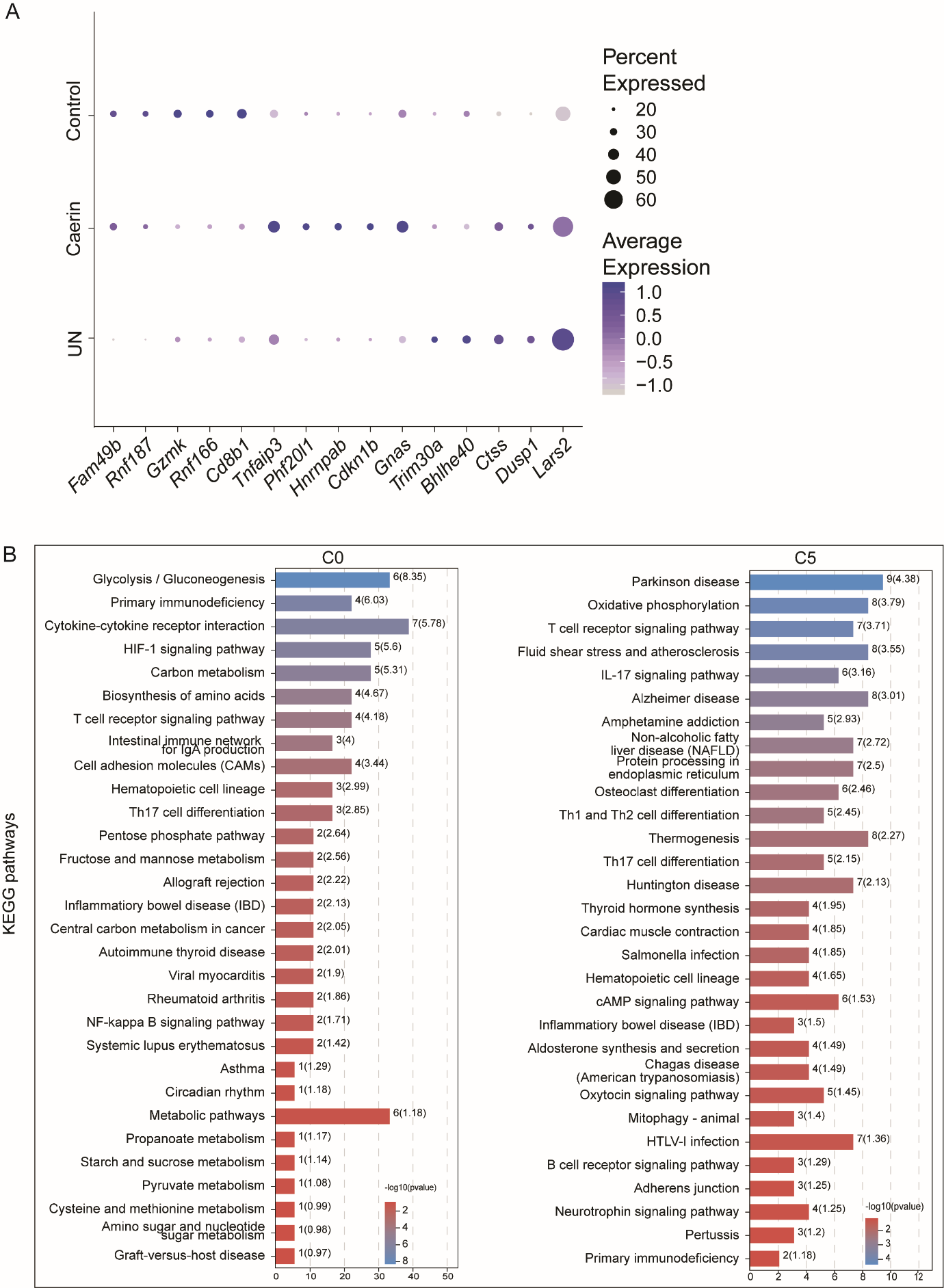


**Figure S7** The bubble map of the top 5 gene differently expressed in NK cell populations of the untreated, caerin and control groups (A). The top 30 KEGG pathways enriched in C0_AcivNK and C5_AdapNK subpopulations (B).


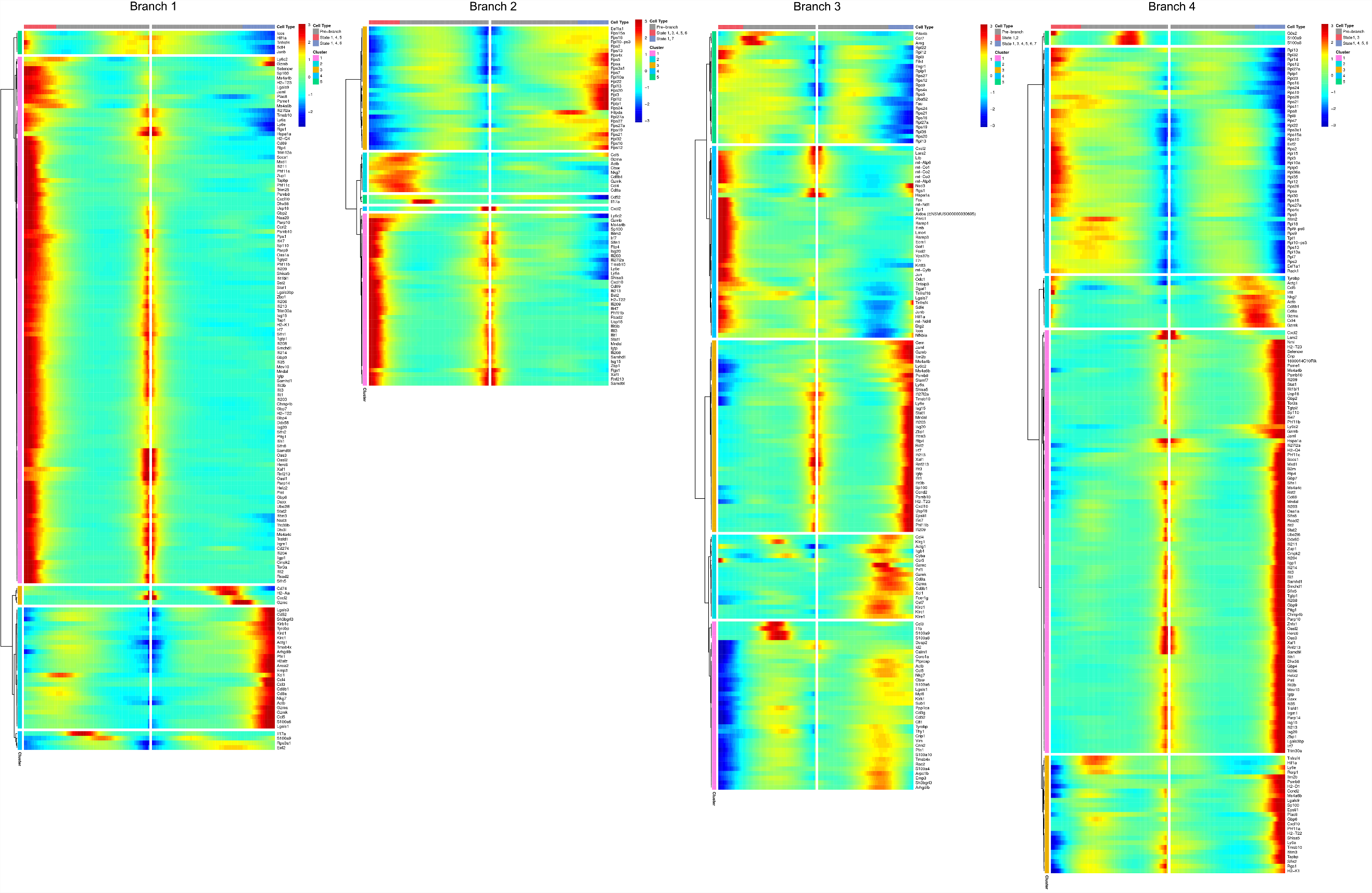
 **­Figure S8** Hierarchy clustering of the gene set with similar expression trend in different branches of the trajectory of NK cells.


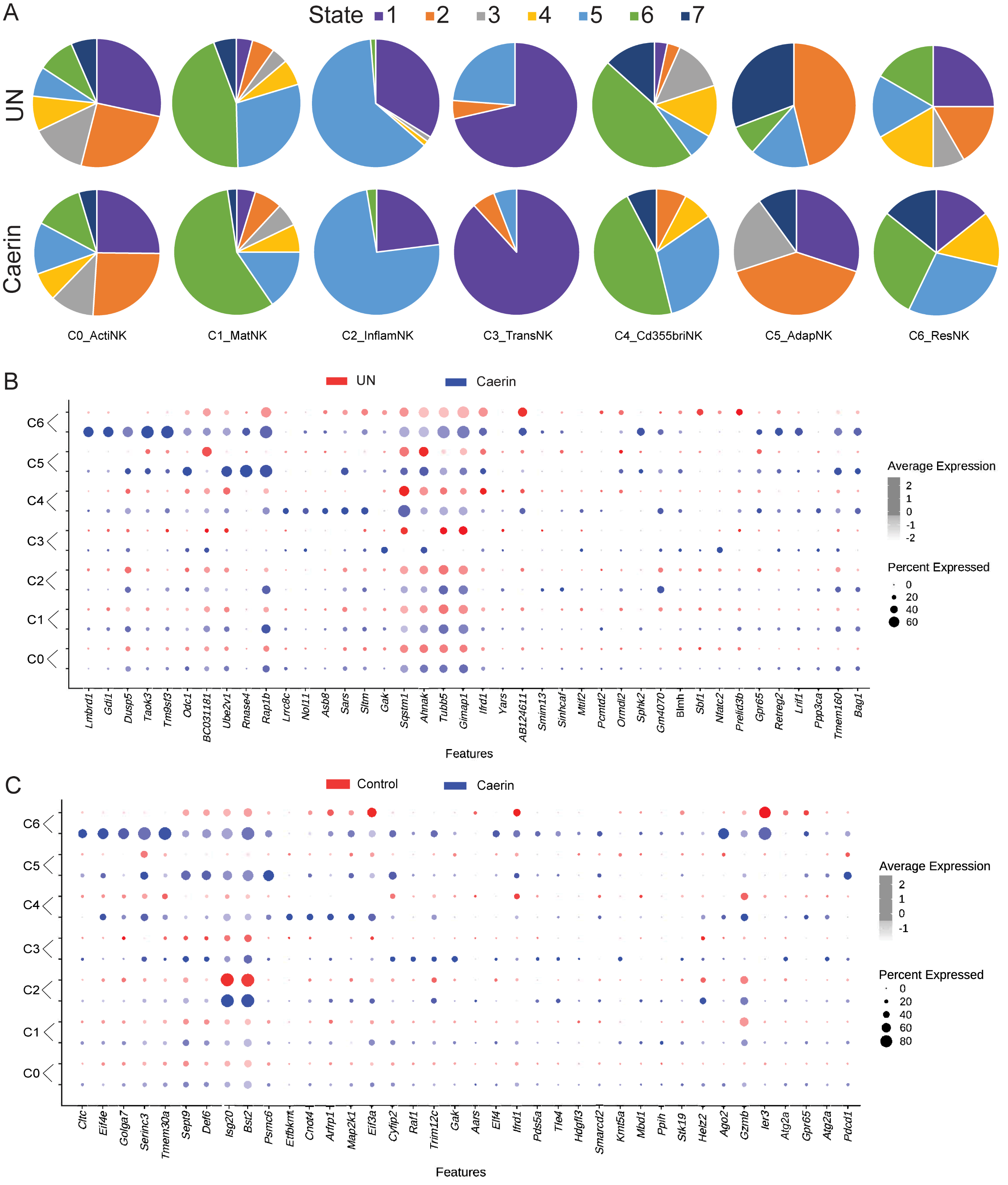


**Figure S9** (A) Composition of the cell states within each NK subpopulation is displayed via pie charts. (B) Comparison of the top 5 genes differentially expressed in different NK subpopulations of the caerin and untreated groups. (C) Comparison of the top 5 genes differentially expressed in different NK subpopulations of the caerin and control groups.


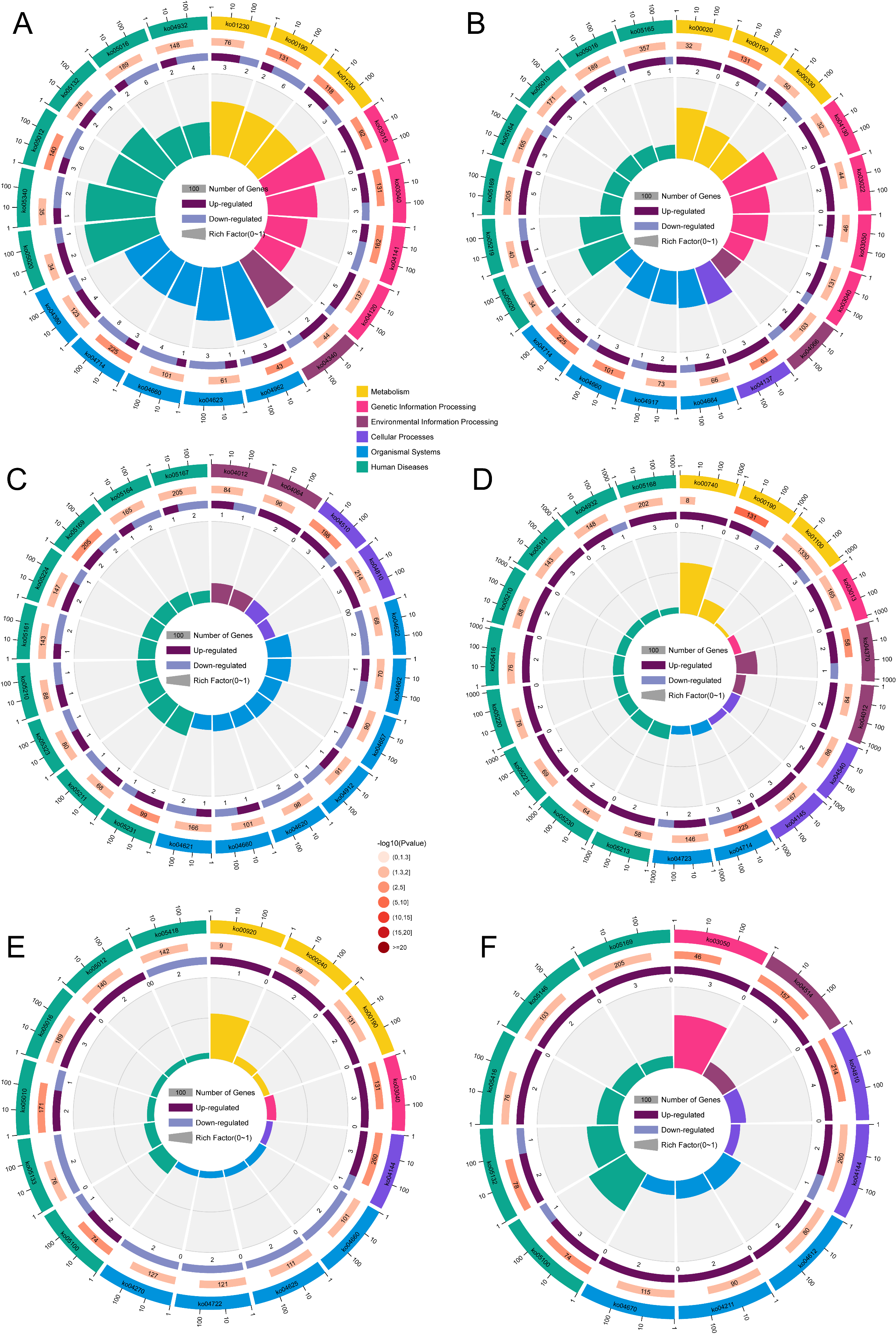


**Figure S10**_­_ The KEGG pathways enriched (*P* < 0.05) in the subpopulations of the caerin group in comparison to the control group: (A) C1_MatNK, (B) C2_InflamNK, (C) C3_TransNK, (D) C4_Cd355briNK, (E) C5_AdapNK and (F) C6_ResNK. The classifications and the IDs of KEGG pathways are shown, as well as the numbers of significantly upregulated and downregulated genes of each KEGG pathways are shown.


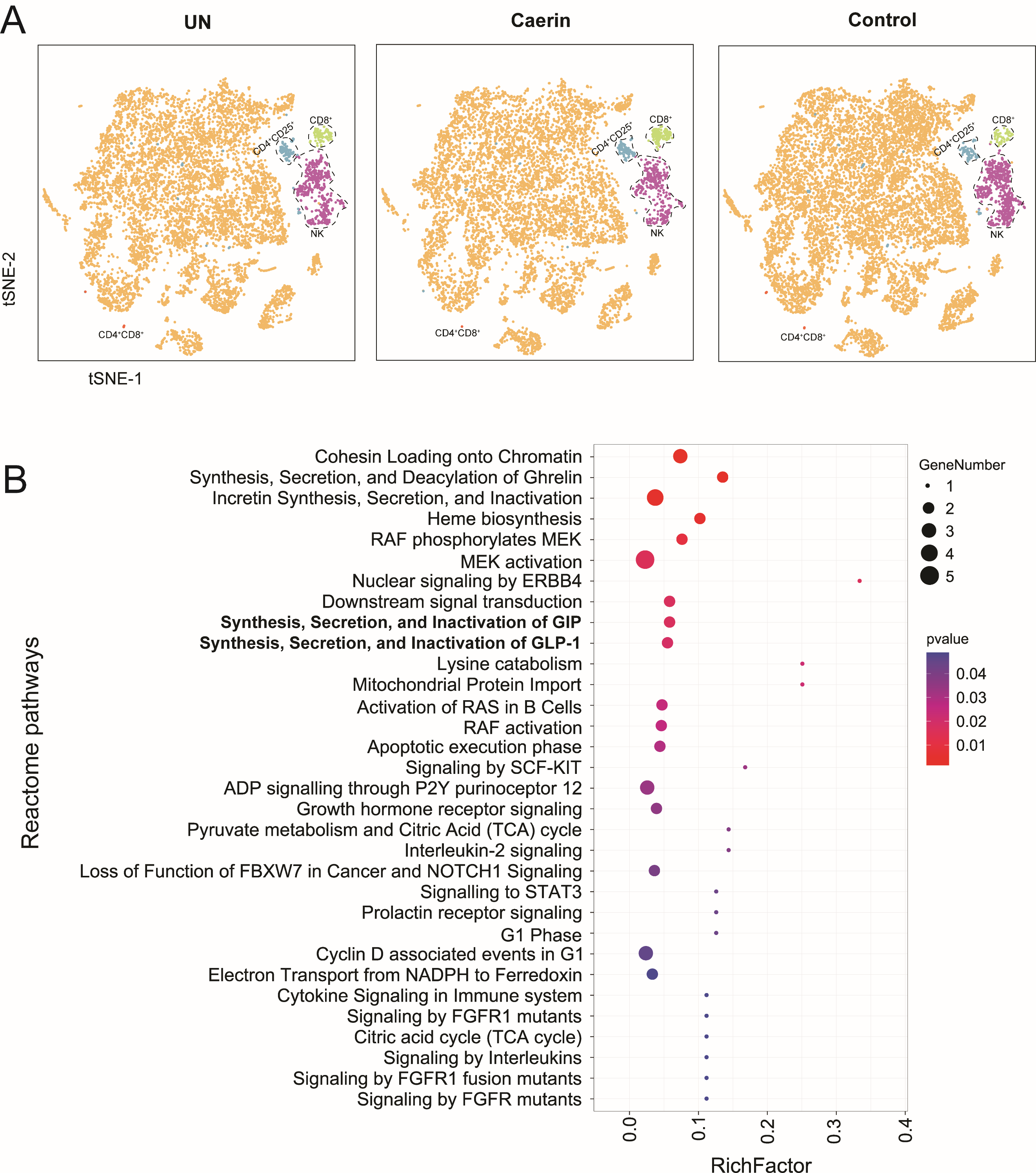


**Supplementary Figure S11** (A) 2D visualisation of cell populations inferred from RNA-seq data for all CD45^+^ cells in untreated and treated with caerin or control gel; T cell populations (including CD8^+^, CD4^+^CD8^+^ and CD4^+^CD25^+^) and NK cells were featured, and colour coded as indicated. (B) Reactome pathways enriched by the unique marker genes of caerin group.


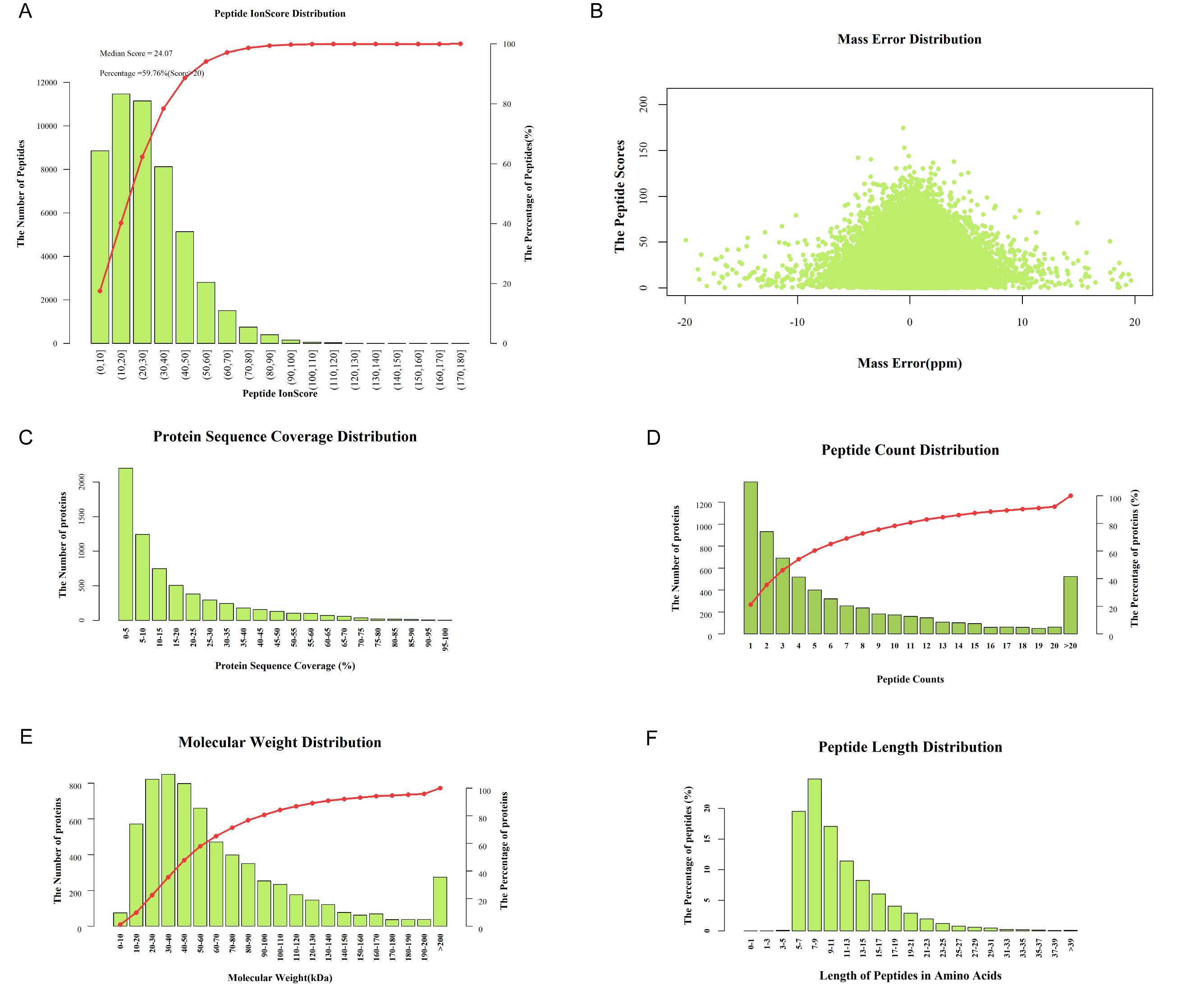


**Figure S12** Summary of quality control (QC) of the quantitative proteomic analysis of tumour tissues, (A) peptide ionScore distribution, (B) mass error distribution, (C) protein sequence coverage distribution, (D) peptide count distribution, (E) molecular weight distribution and (F) peptide length distribution.


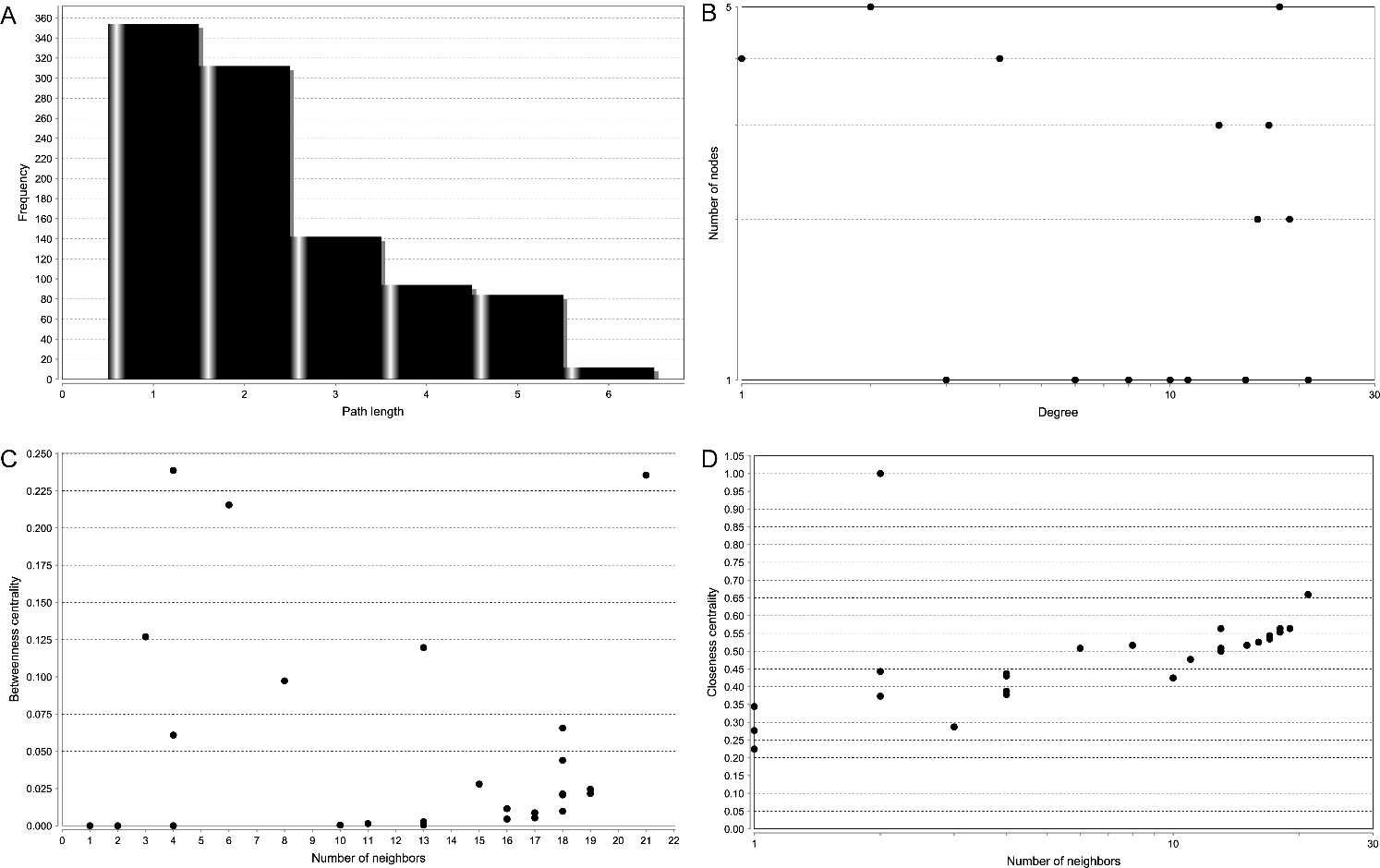


**Figure S13** Statistical analysis of the PPIs shown in **Figure 7C**, (A) shortest path length distribution, (B) node degree, (C) betweenness centrality and (D) closeness centrality.

# Supplementary Methods

## Single cell RNA sequencing method and data process

## GEM generation and barcoding

The Single Cell 3’ Protocol upgraded short read sequencers to deliver a scalable microfluidic platform for 3’ digital gene expression profiling of 500–10,000 individual cells per sample. Libraries were generated and sequenced from the cDNA and the 10x Barcodes were used to associate individual reads back to the individual partitions. Incubation of the GEMs produced barcoded, full-length cDNA from poly adenylated mRNA. After incubation, the GEMs were broken and the pooled fractions were recovered. Full-length, barcoded cDNA was amplified by PCR to generate sufficient mass for library construction.

## Library construction and sequencing

R1 (read 1 primer sequence) were added to the molecules during GEM incubation. P5, P7, a sample index, and R2 (read 2 primer sequence) were added during library construction via End Repair, Atailing, Adaptor Ligation, and PCR. The final libraries contained the P5 and P7 primers used in Illumina bridge amplification. The Single Cell 3’ Protocol produced Illumina-ready sequencing libraries. A Single Cell 3’ Library comprised standard Illumina paired-end constructs which began and ended with P5 and P7. The Single Cell 3’ 16 bp 10x Barcode and 10 bp UMI were encoded in Read 1, while Read 2 was used to sequence the cDNA fragment. Sample index sequences were incorporated as the i7 index read. Read 1 and Read 2 were standard Illumina® sequencing primer sites used in paired-end sequencing.

## Genome Alignment

Cell Ranger (http://support.10xgenomics.com/single-cell/software/overview/welcome) was used in conjunction with STAR (https://github.com/alexdobin/STAR). A read was considered exonic if at least 50% of it intersects an exon, intronic if it was non-exonic and intersects an intron, and intergenic if otherwise. Cell Ranger further aligned exonic reads to annotated transcripts to verify compatibility. A read compatible with the exons of an annotated transcript, and aligned to the same strand, was considered mapped to the transcriptome. Only reads that were confidently mapped to the transcriptome were used for UMI counting.

## Expression QC

Seurat was used to explore QC metrics and filter cells with the gene counts between 500 and 4000 per cell, UMI counts below 8000 per cell and the percentage of mitochondrial genes lower than 10%. Based on the number of genes identified, total UMI number and the ratio of the mitochondria gene expression in one cell, high quality cells were filtered to be included in the following subtype clustering (**Figure S1**). With the quality control filters, 4,648 cells (untreated tumour), 6,523 cells (caerin 1.1/1.9 treatment) and 6,409 cells (the negative control P3 treatment) were included in the analysis (**Supplementary Data 1**).

## Clustering cells and differentially expressed genes analysis

Seurat approach was used and distances between the cells were calculated. Modularity optimisation techniques SLM(Blondel et al., 2008) was applied to iteratively group cells together to optimise the standard modularity function. The likelihood-ratio test was used to find differential expression for a single cluster, compared to all other cells. Differentially expressed genes were identified with *P*-value ≤ 0.01, Log FC ≥ 0.360674, where LogFC means log fold-change of the average expression between the two groups, and the percentage of cells where the gene is detected in specific cluster > 25%.

## Constructing single cell trajectories, differential expression analysis

Single cell trajectory was analysed using a matrix of cells and gene expressions by Monocle (Version 2.6.4). Monocle reduced the space down to one with two dimensions and ordered the cells (sigma = 0.001, lambda = NULL, param.gamma = 10, tol = 0.001). The trajectory in the reduced dimensional space was visualised. Monocle found genes differentially expressed between groups of cells and assessed the statistical significance of those differences. Key genes related to the development and differentiation processes with FDR<1E-5 were identified and grouped genes with similar trends in expression.

## Sample preparation for quantitative proteomics

## TMT-10plex labelling and high pH reversed-phase fractionation

The samples were homogenised thoroughly in SDT buffer (4% (w/v) SDS, 100 mM Tris-HCl pH 7.6, 0.1M DTT) at 4^o^C and the total protein contents were quantified using the Pierce BCA protein assay on a NanoDrop 2000 (Thermo Fisher Scientific, Bremen, Germany). Tryptic peptides were desalted on Sep-Pak C18 columns (Waters, Milford, MA) and lyophilised. The samples containing 100 μg peptides were labelled by TMT10-plex following the manufacturer’s instruction. In this study, 126, 127N and 127C were used to label untreated tumour samples; 128N, 128C and 129N were used to caerin group samples; and the control group samples were labelled using 129C, 130N and 130C. The labelled samples were mixed and fractionated using a Pierce^TM^ high pH Reversed-Phase Peptide Fractionation Kit (Thermo Fisher Scientific, US) and lyophilised on a SpeedVac and resuspended 12 μL 0.1% formic acid for LC-MS/MS analysis.

## Easy nLC tandem Q-Exactive MS/MS analyses

Aliquot of 10 μl from each sample solution were loaded onto a two dimensional EASY-nLC1000 system coupled to The samples were first injected into the sample loading column (Thermo Scientific Acclaim PepMap100, 100 μm × 2 cm, nanoViper C18) and fractionated by the analytic column (Thermo scientific EASY column, 10cm, ID75μm, 3μm, C18-A2) Thermo EASY SC200 trap column (RP-C18, 3 μm, 100 mm × 75 μm). Mobile phase A solution consisted of 0.1% formic acid in water and mobile phase B solution consisted of 0.1% formic acid in 84% acetonitrile. The columns were rinsed with 100% mobile phase B for 8 min and re-equilibrated to the initial conditions for 12 min. The flow rate of the above procedures was 300 nl/min. The ion spray voltage was set to 5500 V, the declustering potential was set to 100 V, the curtain gas flow was set at 30, ion source gas 1 was set at 40, the ion source gas 2 was set at 50 and spray temperature was set at 450^°^C. The mass spectrometer acquired the mass spectral data in an Information Dependant Acquisition, IDA mode. Full scan MS data was acquired over the mass range 300-1800 with a resolution of 70,000 at 200 m/z. AGC (Automatic gain control) target was set at 10^6^, the maximum IT was set at 50ms and the dynamic exclusion was set at 60.0s. In every full scan, twenty MS/MS spectra were obtained. The MS/MS activation type was HCD and the isolation window was 2 m/z. The resolution of MS/MS was 35,000 at 200 m/z, the normalised Collision Energy was set at 30 and the underfill was set at 0.1%.

## Protein identification and quantification

The MS/MS data was searched against the Swissprot Mouse (76,413 sequences, downloaded on Dec 12, 2014) database for protein identification using Mascot 2.2 (Matrix Science, London, UK) and Proteome Discoverer1.4 software (Thermo Fisher Scientific, Waltham, MA, USA) with the following search settings: enzyme trypsin; two missed cleavage sites; precursor mass tolerance 20 ppm; fragment mass tolerance 0.1 Da; fixed modifications: Carbamidomethyl (C), TMT 10plex (N-term),TMT10 plex (K); variable modifications: oxidation (M) , TMT 10plex (Y). The results of the search were further submitted to generate the final report using a cut-off of 1% FDR on peptide levels and only unique peptides were used for protein quantitation. All peptide ratios were normalised by the median protein ratio, and the median protein ratio was 1 after the normalisation. The protein showing a fold change≥1.2 (upregulation≥ 1.2 or downregulation ≤0.83) compared to the untreated group and the *P*-value<0.05 were considered significantly regulated by the treatment and included in further analysis.

## References

Blondel VD, Guillaume J-L, Lambiotte R, Lefebvre E. Fast unfolding of communities in large networks. J Stat Mech 2008;2008(10):P10008.
